# Last-percent improvement in eligibility rates of crop seeds based on quality evaluation using near-infrared imaging spectrometry

**DOI:** 10.1371/journal.pone.0291105

**Published:** 2023-09-20

**Authors:** Osamu Matsuda, Yoshinori Ohara

**Affiliations:** 1 Department of Biology, Faculty of Science, Kyushu University, Fukuoka, Japan; 2 Tokita Seed Co., Ltd., Saitama, Japan; Huazhong Agriculture University, CHINA

## Abstract

As the world population continues to grow, the need for high-quality crop seeds that promise stable food production is increasing. Conversely, excessive demand for high quality is causing “seed loss and waste” due to slight shortfalls in eligibility rates. In this study, we applied near-infrared imaging spectrometry combined with machine learning techniques to evaluate germinability and paternal haplotype in crop seeds from 6 species and 8 cultivars. Candidate discriminants for quality evaluation were derived by linear sparse modeling using the seed reflectance spectra as explanatory variables. To systematically proceed with model selection, we defined the sorting condition where the recovery rate of seeds matches the initial eligibility rate (*iP*) as “standard condition”. How much the eligibility rate after sorting (*P*) increases from *iP* under this condition offers a reasonable criterion for ranking candidate models. Moreover, the model performance under conditions with adjusted discrimination strength was verified using a metric “relative precision” (*rP*) defined as (*P–iP*)/(1*–iP*). Because *rP*, compared to precision (= *P*), is less dependent on *iP* in relation to recall (*R*), i.e., recovery rate of eligible seeds, the *rP-R* curve and area under the curve also offer useful criteria for spotting better discriminant models. We confirmed that the batches of seeds given higher discriminant scores by the models selected with reference to these criteria were more enriched with eligible seeds. The method presented can be readily implemented in developing a sorting device that enables “last-percent improvement” in eligibility rates of crop seeds.

## Introduction

The world population continues to grow, reaching 8 billion in 2022, and is expected to reach 8.5 billion in 2030 and 9.7 billion in 2050 [1]. The issue of stable production and supply of food is thus becoming more and more important for sustainability of human society. Developing high-yielding cultivars and spreading them around the world would be one of the effective measures to address this issue. However, no matter what cultivar is used for crop production, the superior traits of that cultivar will not be exhibited without high-quality seedlings. Since ancient times in Japan, there has been a term “nae-hansaku”, meaning that “half of the growth is determined by the seedlings”. Taking further, high-quality seedlings also cannot be prepared in the absence of high-quality seeds. Hence, how to produce and prepare high-quality seeds, how to store them without deteriorating that quality, and how to evaluate the current state of their quality are urgent technical challenges that humankind should face in cooperation.

The importance of seed quality control is strongly recognized by national and international public institutions. International seed testing association (ISTA) provides guidelines on how to test seed quality for almost all major crop species [2]. The Japanese government is enforcing a law “Plant Variety Protection and Seed Act”, which stipulates the quality standards to be met by seeds for crop production when distributed on the market. One of the reasons for such public oversight is that the quality of seeds, especially in terms of germinability and genetic purity, is usually hard to identify by appearance or other simple criteria. Accidental spread of low-quality seeds could lead to not only local food shortages, but also impoverishment of farmers.

While public quality control certainly helps prevent seed-induced crop failures, there are always two sides to everything. If the eligibility rate that seeds must meet was set at, e.g., 90%, even seed lots with an eligibility rate of 89% lose their commercial value. In reality, fierce quality competition among seed suppliers renders even legal standards uncompetitive. As a result, tons of seeds with no commercial value are being generated despite their potential to contribute to crop production. This can be called a merciless “seed loss and waste”.

Even for major crop species, the eligibility rate of seeds does not necessarily exceed the legal standard from the post-harvest, unsorted state. The occurrence of ineligible seeds is due in part to seed maturation failure in the mother plant, while in part to damage, deterioration, or contamination during the process of post-harvest seed preparation. A high eligibility rate within a reach of legal standards cannot be safely achieved without passing through a multi-stage sorting. Large-scale devices used for this purpose include spiral, trommel, vibratory, magnetic, gravity, and color separators [3, 4]. Nevertheless, if the eligibility rate falls below the target, the seeds prepared through sowing, cultivation, harvesting, and sorting will become nothing more than a pile of waste. From this point, there is usually no way to improve the eligibility rate even last percent.

The above-mentioned devices for seed sorting are based on the premise that the quality of seed is reflected in features detectable from its outer surface or exterior. These include shape, surface texture and stickiness, specific gravity, and color. The devices are suitable for mass sorting, allowing most of the harvested seeds to be prepared in high quality. By contrast, they have the drawback that it is difficult to fine-tune their operating conditions. Moreover, looking at the biochemical properties of seeds, alterations in the hidden embryo or endosperm than those in the exposed seed coat have a far greater impact on seed function. For example, oxidation of endosperm lipids or feeding of embryos by specific pests does not immediately give rise to changes in the appearance or weight of seeds, but does impair their germinability. It is presumed that “seed loss and waste” is caused by ineligible seeds of this kind.

Near-infrared (NIR) spectrometry is an effective technique for non-destructive detection of differences and changes in chemical composition in substances mainly composed of organic compounds. In other words, it has a high affinity with food, pharmaceuticals, and agricultural products, and so its application for their quality control is expanding [5]. Imaging spectrometry, which has evolved primarily in the field of remote sensing, is another technique gaining attention in the quality control of these non-homogenous commodities. The fusion of these emerging techniques, i.e., NIR imaging spectrometry, is highly expected to provide clues to abolish “seed loss and waste”. Indeed, there are increasing reports of the application of NIR imaging spectrometry in evaluating seed quality in terms of viability, vigor, genetic purity, and the presence or absence of insect and fungal infestations [6–9]. However, the fact that “seed loss and waste” still continues to occur seems to testify that the technique has yet to reach a practical phase, or its social implementation has not progressed smoothly. One of the factors being pointed out as an impediment to the spread of this technique is high cost of the equipment required, NIR multi- or hyperspectral cameras [9]. Given their active use in the food industry [10], however, this should not be the root cause. Though there is a difficulty in that handling of this technique requires multidisciplinary expertise, the time has come that individuals and teams with such aptitude in the field of seed science and industry should set out to consistent activities from research to practical application.

It may be just one of many attempts around the world, we are aiming to develop a sorting device based on NIR imaging spectrometry to abolish “seed loss and waste”. Here we demonstrate a practical workflow from the occurrence of an ineligible seed lot to the start of sorting by the envisioned device, which will pave the way to improve the eligibility rate of seeds to the last percent.

## Materials and methods

### Seed materials

Seeds were prepared and provided by Tokita Seed Co., Ltd. Japan. Information for all seed materials used in this study, including harvest locations and years, is summarized in Table 1.

**Table 1:** Seed materials used in this study.

Family	Genus	Species	Cultivar	Origin		Lot	Batch	Quality Breakdown						
								Germination*^1^			Haplotype			Total
				Location	Year			Normal	Abnormal	Dead	F1	Inbred	ND*^2^	
Cucurbitaceae	*Cucurbita*	Squash	5	China	2020	A	1	194	17	29	–	–	–	240
		(*C*. *maxima* × *moschata*)					2	128	25	31	–	–	–	184
Fabaceae	*Pisum*	Pea	19	Japan	2022	A	1	259	47	93	–	–	–	399
		(*P*. *sativum*)		(Ehime)			2	156	28	129	–	–	–	313
Asteraceae	*Lactuca*	Lettuce	803	China	2020	A	1	345	58	8	–	–	–	411
		(*L*. *sativa*)					2	515	63	19	–	–	–	597
Amaryllidaceae	*Allium*	Bunching Onion	22	France	2021	A	1	363	58	58	–	–	–	479
							2	337	77	96	–	–	–	510
		(*A*. *fistulosum*)	51	Turkey	2018	A	1	390	71	19	–	–	–	480
Solanaceae	*Solanum*	Tomato	94	Thailand	2015	A	1	254	16	66	–	–	–	336
						B	1	272	29	35	–	–	–	336
		(*S*. *lycopersicum*)	221	Japan (Saitama)	2018	A	1	157	50	125	–	–	–	332
Brassicaceae	*Brassica*	Cauliflower	47	China	2017	A	1	415	46	11	351	91	30	472
							2	494	52	27	418	120	35	573
		(*B*. *oleracea var*. *botrytis*)			2020	B	1	413	94	39	404	75	67	546

*^1^ “Abnormal” denotes seeds that did not grow into healthy seedlings after emergence, while “Dead” those not germinated within the test period.

*^2^ Not determined because tissue extracts could not be prepared from dead or abnormally-germinated seeds.

### Near-infrared hyperspectral imaging

NIR hyperspectral images of seeds were captured using an imaging system composed of a push-broom NIR hyperspectral camera (CV-N801HS, Sumitomo Electric Industries Ltd., Japan), a NIR lens with a focal length of 30 mm (Sumitomo Electric Industries Ltd.), and a motorized shutter device (Lambda SC + IQ35-SA *Smart*Shutter, Sutter Instrument Company, CA, USA) attached in front of the lens. Illumination was supplied through a facing pair of anhydrous synthetic quartz-transmission lights (PDL-S-250VAB, NIPPON P∙I Co., Ltd., Japan) placed adjacent to DC halogen lamps with aluminum reflector (PLL-250/AL, NIPPON P∙I Co., Ltd.). Seeds were aligned on shallow wells (1-mm depth) of microplate-formatted (24, 48 or 96 wells) matt black-finished custom polyvinyl chloride (PVC) trays. They were transferred by a stepping motor-driven (RKS545AAD-FC30LA-1, Oriental Motor Co., Ltd., Japan) conveyor device (CSSK50-T-100-100, NKE Corporation, Japan). Images were taken twice with the trays rotated 180°, in preparation for mutual outer cross-validation in the subsequent discriminant modeling. The camera was equipped with a 16-bit cooled indium-gallium-arsenide and gallium-arsenide-antimonide (InGaAs/GaAsSb) type-II super lattice (T2SL) focal-plane array (FPA), having sensitivity in the wavelength range of 950–2,350 nm, and was capable of acquiring 320 pixel-wide and 256 spectral-band line image at a single exposure. As the pixel size of the FPA was 30×30 μm and the horizontal field-of-view (FOV) was set to 89.6 mm by applying a working distance of 280 mm, the resulting images had spatial resolution of *ca*. 90 ppi with a spectral sampling pitch of 6 nm. Brightness of the images was calibrated to absolute reflectance by linear interpolation using an image of the 99% reflectance area of a multistep diffuse reflectance standard (SRT-MS-050, Labsphere Inc., NH, USA) and a darkfield image (0% reflectance).

### High-resolution imaging of seed appearance

A portion of the seeds that underwent NIR hyperspectral imaging was subjected to high-resolution appearance photography using an imaging system composed of an 8k color CMOS line-scan camera (EV71YC4CCL8005-BA0, Teledyne e2v Ltd., CA, USA), 0.4× object-side telecentric lens (MPHC-04F-150, Optart Corporation, Japan), and a pair of high-uniformity white-LED bar lights (IDBA-HMS150WHV-S, Leimac Ltd., Japan). Seeds were placed in KingFisher 96 microplates (Thermo Fisher Scientific Inc., MA, USA) or aligned on shallow wells (1-mm depth) of microplate-formatted (24, 48 or 96 wells) matt black or white-finished custom PVC trays. They were transferred by a stepping motor-driven (RKS564AAD-TS30-1, Oriental Motor Co., Ltd.) conveyor device (GVHA-100-1000-6-NV-NM-NH-D-R, MISUMI Group Inc., Japan). As the pixel size of the line sensor was 5×5 μm, the resulting images had spatial resolution of 2,032 ppi. White balance correction of the images was carried out by using appropriate reflectance areas of the multistep diffuse reflectance standard (SRT-MS-050, Labsphere Inc.).

### Germination test and haplotype analysis

After NIR hyperspectral and/or high-resolution imaging, seeds were subjected to germination tests as prescribed by ISTA [2]. In brief, seeds were sown on a sheet of moist filter paper laid in a plastic container (lettuce, bunching onion, tomato and cauliflower) or on soil (squash and pea), and incubated until the germination deadline under light and temperature conditions suitable for each crop species: continuous light at 20°C for 6 d (lettuce) or 9–10 d (bunching onion); 16/8 h-light and dark photoperiod at 30/20°C for 5 d (cauliflower) or 10–11 d (tomato); greenhouse under natural daylength where the temperature was within the range of 20–30°C for 7–8 d in summer (squash), or 12–22°C for 13–16 d in fall to winter (pea). No pretreatment for germination was applied in this study.

The cauliflower cultivar No. 47 is premised to be used as an intraspecific F1 hybrid. Due to relaxation of self-incompatibility in female flowers that occurs as their lifetime approaches, it has yet to be achieved in this cultivar to completely prevent formation of undesired inbred seeds. To clarify whether each cauliflower seed was an F1 hybrid or inbred, paternal haplotype was determined by zymogram banding patterns of phosphoglucomutase isozymes [11] extracted from seedlings post-germination.

### Discriminant modeling for seed quality evaluation

Datasets prepared through the analysis of each seed batch in Table 1 are summarized in Table 2. A schematic of the following procedures is shown in S1 Fig.

**Table 2:** Datasets prepared through the analysis of each seed batch.

Seed Material				Dataset						
Species	Cultivar	Lot	Batch	Symbol	Focused Trait*^1^	Eligibility		Total	*iP*	
						OK	NG			
Squash	5	A	1	d-Sq5A1	G	194	46	240	80.8%	
			2	d-Sq5A2	G	128	56	184	69.6%	*^2^
Pea	19	A	1	d-Pe19A1	G	259	140	399	64.9%	
			2	d-Pe19A2	G	156	157	313	49.8%	*^2^
Lettuce	803	A	1	d-Le803A1	G	345	66	411	83.9%	
			2	d-Le803A2	G	515	82	597	86.3%	
Bunching Onion	22	A	1	d-Bo22A1	G	363	116	479	75.8%	
			2	d-Bo22A2	G	337	173	510	66.1%	*^2^
	51	A	1	d-Bo51A1	G	390	90	480	81.3%	
Tomato	94	A	1	d-Tm94A1	G	254	82	336	75.6%	
		B	1	d-Tm94B1	G	272	64	336	81.0%	
	221	A	1	d-Tm221A1	G	157	175	332	47.3%	
Cauliflower	47	A	1	d-Ca47gA1	G	415	57	472	87.9%	*^3^
				d-Ca47hA1	H	351	91	442	79.4%	*^4^
				d-Ca47A1	G+H	329	143	472	69.7%	
			2	d-Ca47gA2	G	494	79	573	86.2%	*^3^
				d-Ca47hA2	H	418	120	538	77.7%	*^4^
				d-Ca47A2	G+H	382	191	573	66.7%	
		B	1	d-Ca47gB1	G	413	133	546	75.6%	*^3^
				d-Ca47hB1	H	404	75	479	84.3%	*^4^
				d-Ca47B1	G+H	342	204	546	62.6%	

*iP*, initial precision (eligibility rate before sorting).

*^1^ G and H denote germinability and paternal haplotype, respectively.

*^2^ Values differ from the population mean because of preferred sampling for low-ranked (estimated as ineligible) seeds.

*^3^ Seeds germinated normally are counted as “OK” regardless of their paternal haplotype.

*^4^ F1 hybrid seeds are counted as “OK” regardless of their germinability.

#### Dataset preparation

NIR reflectance spectra (***R***) of individual seeds were obtained by averaging the spectra recorded in seed-occupied pixels in the NIR hyperspectral image. Eleven additional spectra (***X***_2_ to ***X***_12_) were derived from ***R*** (= ***X***_1_) by applying reciprocal (***R***^-1^) [12, 13] or pseudo-absorbance (–log***R***) [14] transformation aimed at improving linearity, in combination with or without standard normal variate (SNV) transformation for baseline correction and/or Savitzky-Golay (SG) smoothing filter [15] for denoising (S1 Fig, step 1). SNV transformation was carried out using a valid spectral range defined as 980–2,200 nm (corresponding to 201 wavebands). SG filter was applied with 3rd-order polynomial and a filter length of 5. All of 12 spectra were examined as explanatory variables for predicting quality traits of individual seeds.

For training discriminant models, the quality trait of seeds to predict (objective variable ***y***) was assigned with a dummy variable: 1 for seeds that were later clarified as eligible and −1 for others (S1 Fig, step 1). For prediction of germinability, seeds normally germinated were assigned with 1, while those germinated abnormally, i.e., the ones that did not grow into healthy seedlings after emergence, and non-germinated seeds, that we regarded as dead, with −1. For prediction of paternal haplotype of cauliflower seeds, F1 hybrid and inbred were assigned with 1 and −1, respectively. For direct prediction of fully eligible cauliflower seeds with commercial value, F1 hybrid that germinated normally was assigned with 1, and −1 for others.

#### Derivation of candidate discriminant models

Datasets comprised of ***y*** and each of 12 NIR spectra (***X***_1_ to ***X***_12_) were subjected to discriminant modeling for seed quality evaluation. Models were basically derived by partial least squares discriminant analysis (PLS-DA) [16, 17], but to improve their interpretability and performance, we introduced a step for preselecting explanatory variables (wavebands) by L1 regularization. Specifically, an ordinary PLS-DA model was first derived and let its standard partial regression coefficients (SPRCs) be ***β*** (S1 Fig, step 2). Reciprocal of the absolute values of ***β*** was then set as the penalty factor to execute adaptive least absolute shrinkage and selection operator (LASSO) [18] (S1 Fig, step 3). Finally, the reduced explanatory variable combinations presented in the LASSO solution path were reapplied to PLS-DA to complete a series of candidate discriminant models (S1 Fig, step 4). Scripts and datasets for replicating these procedures are provided as S1 File. How to select superior models from the candidates is one of the subjects of this study, and so will be addressed in the Results section.

### Software

Software for operating the NIR hyperspectral and 8k line-scanning camera systems, and for analyzing hyperspectral images was developed in managed C# code using Microsoft Visual Studio Community edition (version 2022, https://visualstudio.microsoft.com/) with extensions Emgu CV (version 4.7.0, https://www.emgu.com/), a.NET wrapper to the OpenCV image processing library (version 4.7.0, https://www.opencv.org/) and Extreme Optimization Numerical Library for.NET (version 8.1.19, https://www.extremeoptimization.com/). The software for directly visualizing discriminant scores of seeds within hyperspectral images is provided as S2 File, which works on 64-bit versions of Microsoft Windows operating systems with.NET6 runtime environment. Discriminant modeling with adaptive LASSO and PLS-DA algorithms were performed on R statistical software (version 4.3.1, https://www.r-project.org/) using packages ‘glmnet’ and ‘pls’. Variable importance in projection (VIP) [19, 20] of the resulting PLS-DA models was calculated using an R package ‘plsVarSel’. All graphs were drawn using ‘ggplot2’ and related R packages except the heat maps drawn using RINEARN Graph 3D software (version 5.6.32, https://www.rinearn.com/graph3d/).

## Results

### Appearance and NIR reflectance spectra of eligible and ineligible crop seeds

**Fig 1:**
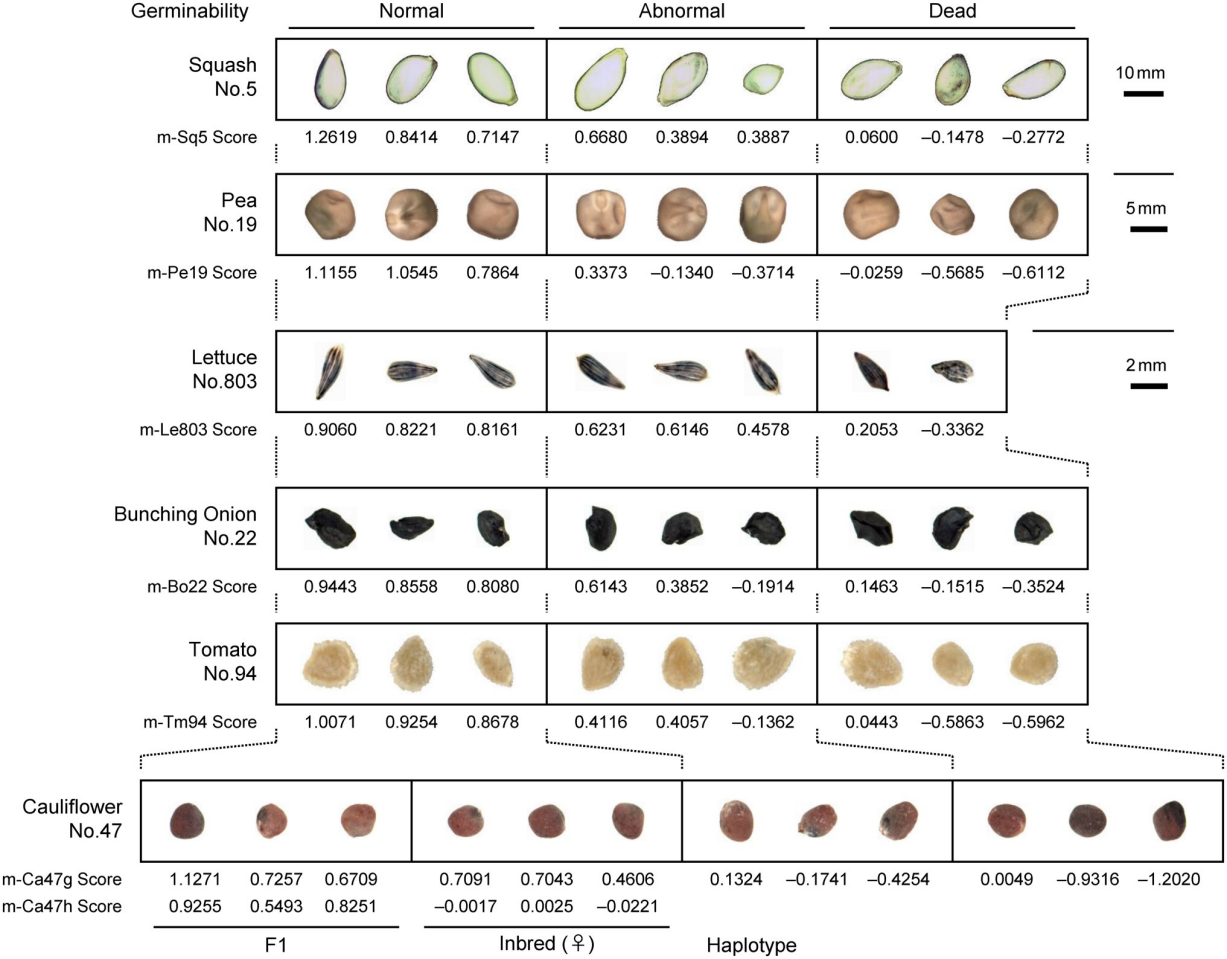
Appearance of eligible and ineligible crop seeds. Seeds were subjected to germination test after capturing NIR hyperspectral and 8k color images. For cauliflower seeds, paternal haplotype was also determined. Images are arranged according to the results of these quality inspections. Numbers below the images represent the discriminant score for each seed given by the corresponding discriminant models in Table 4.

Fig 1 shows high-resolution images of seeds from all crop species used in this study, which are arranged according to their quality as clarified by subsequent germination test and/or haplotype analysis. For all crop species, there were no features in the seed appearance that could guide us to discriminate whether each is eligible or not. In fact, Fig 1 selectively shows eligible and ineligible seeds given distinct high and low scores, respectively, by discriminant models derived later.

**Fig 2:**
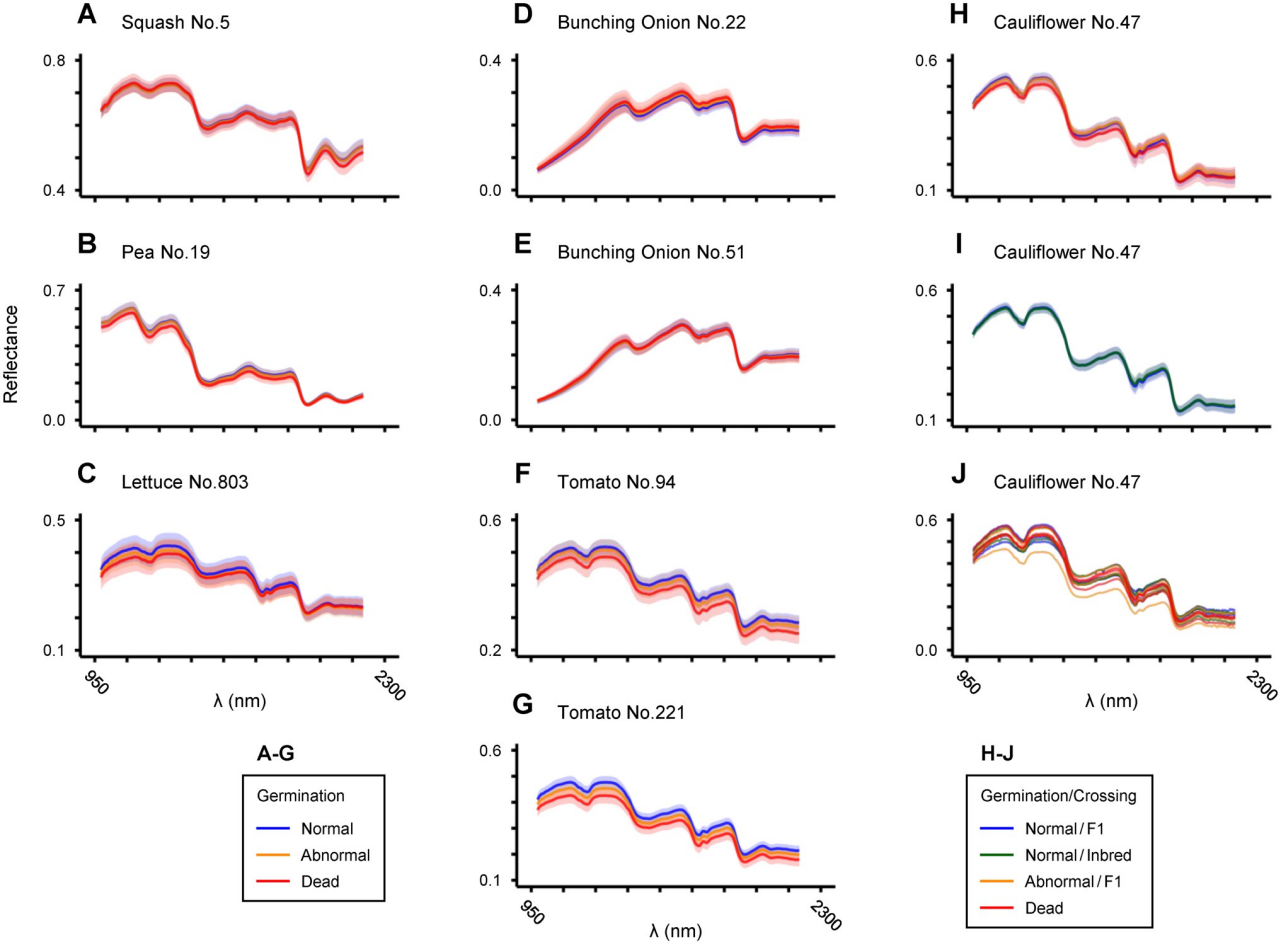
NIR reflectance spectra of eligible and ineligible crop seeds. (A-I) Average (±SD) reflectance spectra of seeds by quality class for the indicated crop cultivars. (J) Reflectance spectra of 5 individual seeds from each quality class in cauliflower cultivar No. 47. The range of X-axis is common to that shown at the bottom (C, G and J).

Fig 2 shows NIR reflectance spectra from eligible and ineligible seeds of each crop species. When the averaged spectra of a large number of seeds were compared, slight but certain differences could be seen in the waveforms between eligible and ineligible classes (Fig 2A–2I). By contrast, when the spectra from individual seeds were compared side by side, it seemed no longer discriminable which waveforms are from the eligible seeds (Fig 2J).

### Defining metrics for discriminant model selection

#### (1) “Standard condition” and related metrics

To predict quality traits of individual seeds from their NIR reflectance spectra with practical accuracy, machine learning techniques were employed to derive discriminant models for seed quality evaluation. What is desired for the models are to give high and low scores, respectively, to seeds in eligible and ineligible classes. But how can we spot the best few among many candidates?

**Fig 3:**
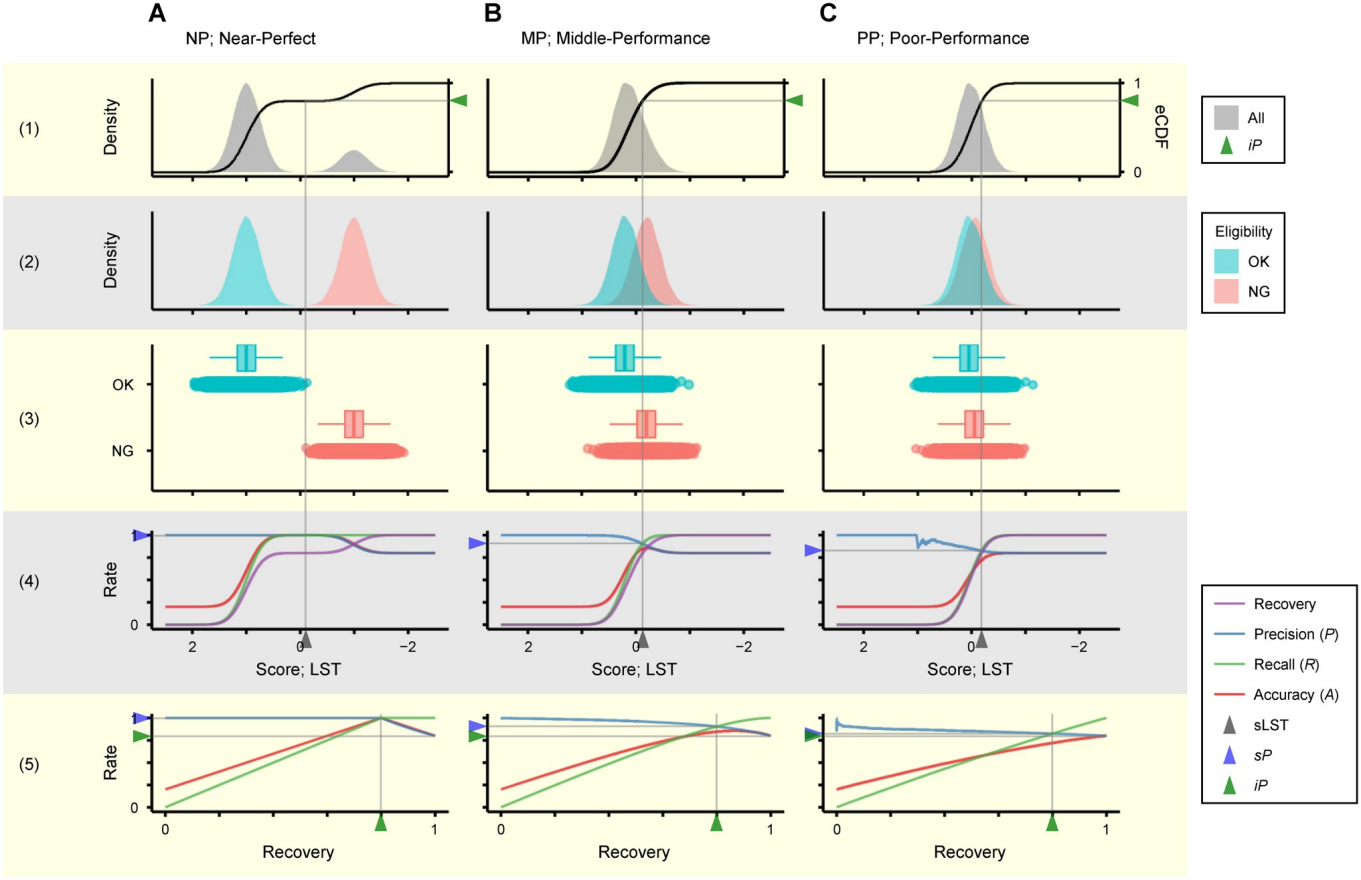
Thought experiments of eligible seed sorting using hypothetical models with different discriminability. Properties of the three hypothetical models NP (A), MP (B) and PP (C) are shown in Table 3. Results expected when these models were applied to a seed batch with 80% initial precision (*iP*, eligibility rate before sorting) are shown. X-axis is common from (1) to (4) representing discriminant score itself, or lower score threshold (LST) set for seed sorting. (1) Density distribution of scores for all seeds (gray filled curves) and empirical cumulative distribution function (eCDF) for seeds above LST (black lines). The latter provides a means to convert between LST and seed recovery rate. (2) Density distribution of scores for seeds in each quality class. Curve heights are scaled by class. (3) Boxplot and scatterplot representations of (2). (4–5) Discrimination performance plotted as a function of LST setpoint (4) or seed recovery rate (5). *sP*, standard precision (eligibility rate after sorting under standard condition); sLST, standard LST (LST that matches seed recovery rate with *iP*).

To find good criteria for model selection, we start with a thought experiment using three hypothetical models with different discriminability: near-perfect (NP), medium- (MP) and poor-performance (PP). For each of eligible and ineligible seeds, the model NP gives scores according to normal distributions *N* (1, 0.25) and *N* (−1, 0.25), MP according to *N* (0.2, 0.25) and *N* (−0.2, 0.25), and PP according to *N* (0.05, 0.25) and *N* (−0.05, 0.25) (Table 3). Fig 3 shows distribution of scores given by each model in case the initial eligibility rate (*iP*, initial precision) was 80% (rows 1–3). Assuming a scheme of eligible seed sorting where seeds are recovered in descending order of score, metrics related to discrimination performance can be plotted as a function of lower score threshold (LST) (row 4) or the recovery rate of seeds (row 5). LST and seed recovery rate are interconvertible, as the latter is trivially derived from the empirical cumulative distribution function (eCDF) of discriminant scores given to individual seeds (row 1). With NP model, precision (*P*), i.e., eligibility rate after sorting, keeps close to 100% until the recovery rate of seeds reaches *iP* (Fig 3A; row 5). With PP model, on the other hand, *P* immediately converges to *iP* (Fig 3C; row 5).

**Table 3:** Thought experiment using hypothetical seed batches and discriminant models.

Seed Batch				Model			Performance					
Eligibility		Total	*iP*	Symbol	Score		Standard*			Overall		
OK	NG				OK	NG	sLST	*sP* (= *sR*)	*sA*	*A*max	AUC-PR	AUC-rPR
52428	13107	65535	80.0%	NP	*N*(1, 0.25)	*N*(–1, 0.25)	-0.1080	100.0%	100.0%	100.0%	1.0000	1.0000
				MP	*N*(0.2, 0.25)	*N*(–0.2, 0.25)	-0.1241	90.3%	84.5%	85.6%	0.9598	0.7991
				PP	*N*(0.05, 0.25)	*N*(–0.05, 0.25)	-0.1859	82.5%	72.0%	80.0%	0.8550	0.2751
39321	26214	65535	60.0%	NP	*N*(1, 0.25)	*N*(–1, 0.25)	-0.0419	100.0%	100.0%	100.0%	1.0000	1.0000
				MP	*N*(0.2, 0.25)	*N*(–0.2, 0.25)	-0.0345	83.0%	79.6%	79.8%	0.9056	0.7640
				PP	*N*(0.05, 0.25)	*N*(–0.05, 0.25)	-0.0555	66.3%	59.6%	61.8%	0.6898	0.2244

*P*, precision (eligibility rate after sorting); *R*, recall (recovery rate of eligible seeds); *A*, accuracy; *iP*, initial *P* (eligibility rate before sorting); LST, lower score threshold; *sP*/*sR*/*sA*, *P*/*R*/*A* under standard condition; *A*max, maximum *A*; AUC-PR/rPR, area under PR/rPR (precision/relative precision-recall) curve.

* Condition where the recovery rate of seeds matches *iP*, and *P* and *R* equalize (*sP* = *sR*).

In real scenes of seed management, *iP* in each seed lot is usually known through small-scale preliminary tests conducted shortly after harvest. What is desired for post-harvest seed sorting is to raise *P* with recall (*R*), i.e., recovery rate of eligible seeds, kept high as much as possible. It is worth noticing in this respect that *P* and *R* equalize when the recovery rate of seeds reaches *iP*, which always holds no matter what discriminant model is used (Fig 3; row 5). We therefore define the condition where the recovery rate of seeds matches *iP* as “standard condition”. Metrics definable under this condition include “standard LST” (sLST) as the lowest score of seeds to be recovered, and “standard precision” (*sP*) as the eligibility rate after sorting (Fig 3; rows 1, 4 and 5). It is clear the better the discrimination performance of the model applied, the higher the *sP* when tested on the same seed batch (Table 3). Below, we may also use the term “standard” to refer to other concepts and metrics under this condition.

#### (2) “Relative precision” and related metrics

Even with PP model, *P* seems adjustable; it increases as the recovery rate of seeds is reduced by raising LST (Fig 3C; rows 4 and 5). If *sP* shortfalls a desired level, discrimination strength should be tightened by raising LST higher than sLST. Then, how can we evaluate the overall performance of discriminant models, including the performance under LST-adjusted “non-standard” conditions?

The performance of discriminant models is often evaluated using ROC (receiver operating characteristics) or PR (precision-recall) curves. In non-defective item selection as with seed sorting, acceptance of ineligible (false-positive) is usually less favorable than rejection of eligible (false-negative). For such purposes, PR curve is preferred over ROC curve due to high sensitivity to false-positive errors [21]. For both curves, it is regarded the larger the area under the curve (AUC), the better the discrimination performance.

The area under the PR curve (AUC-PR), also called average precision, is a useful metric in comparing the performance of multiple discriminant models applied to the same dataset. On the other hand, it has the drawback of being dependent on *iP*, making it unsuitable for verifying if a particular model performs equally on different datasets. To remedy this problem, we introduce “relative precision” (*rP*) defined as:

$$rP\equiv \left\{\;\begin{array}{cc} \frac{P-iP}{1-iP} & \text{if}\;P>iP\\ 0 & \text{otherwise} \end{array}\right.$$ (1)

which is equivalent to *P* scaled to the closed interval of [0, 1]. In connection, we also define rPR curve as the relationship between *rP* and *R*, and area under the rPR curve (AUC-rPR) as an alternative to AUC-PR.

**Fig 4:**
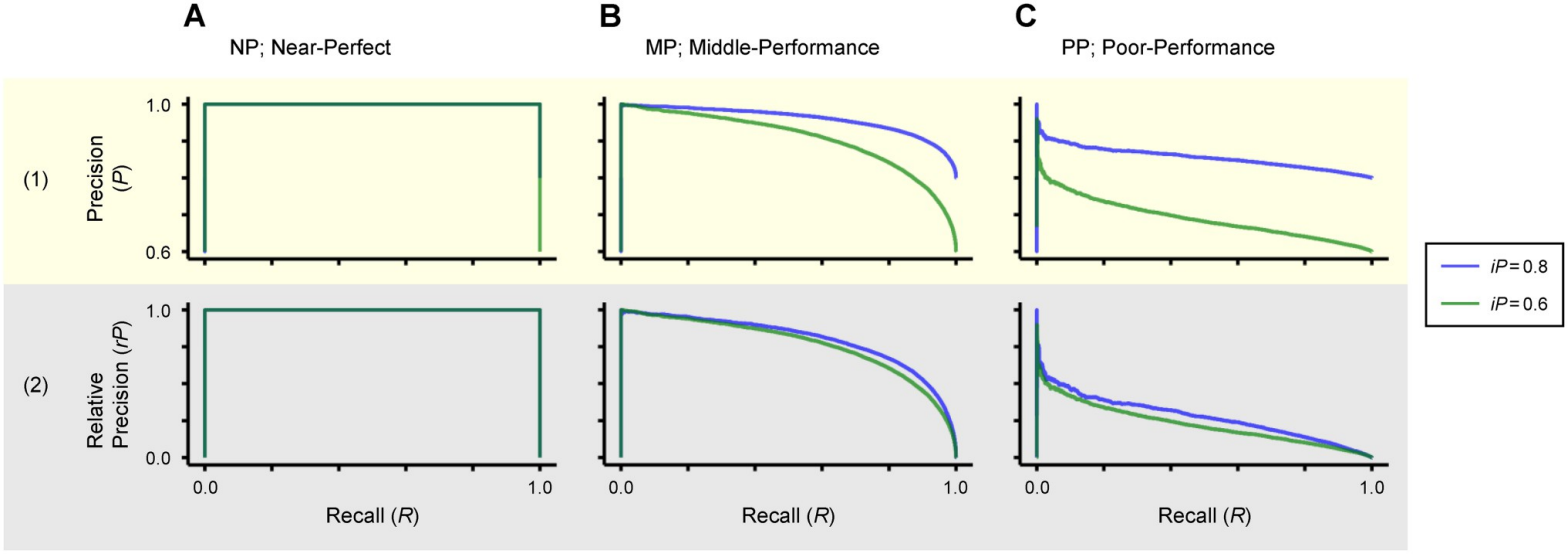
Precision- and relative precision-recall curves drawn by hypothetical models with different discriminability. Properties of the three hypothetical models NP (A), MP (B) and PP (C), and quality breakdown in hypothetical seed batches with initial precision (*iP*, eligibility rate before sorting) of 0.8 (80%) or 0.6 (60%) are shown in Table 3. Relative precision (*rP*) is defines in Eq 1. X-axis in all plots represents recall (*R*, recovery rate of eligible seeds), while Y-axis in (1) and (2) represents precision (*P*, eligibility rate after sorting) and *rP*, respectively.

Fig 4 shows PR and rPR curves for discrimination when individuals in seed batches with 80% or 60% *iP* are scored by the three hypothetical models. Additionally, Table 3 summarizes AUC-PR and AUC-rPR in these cases along with metrics related to standard performance. Though AUC-rPR is not fully independent of *iP*, its effect is far less than on AUC-PR. Accordingly, AUC-rPR reduces the risk of overestimating the performance of poor models when tested on seed batches with higher *iP*. We also paid attention on the difference between standard (*sA*) and maximum accuracy (*A*max) to check if the sorting under standard condition is sufficiently drawing the potential of discriminant models (Table 3). As mentioned, however, what is desired for seed sorting is not maximizing the accuracy (*A*) of discrimination, but raising *P* to the desired level.

### Features of selected discriminant models

#### (1) Effects of waveband selection

While discriminant models can be derived by any algorithm such as PLS-DA, support vector machine (SVM), and even deep learning, PLS-DA has been preferred due to high relative performance and interpretability of the models. Yet, PLS-DA models with non-zero partial regression coefficients (PRCs) for hundreds of explanatory variables (wavebands) are still too complex to grasp the basis for discriminability. We therefore introduced a step for variable selection before executing PLS-DA to reduce the number of wavebands incorporated in the models. As this procedure is somewhat specific to this study, we evaluated how it affected on the structure and performance of the resulting PLS-DA models. They were basically selected for being frequently high-ranked in iterative derivation.

**Fig 5:**
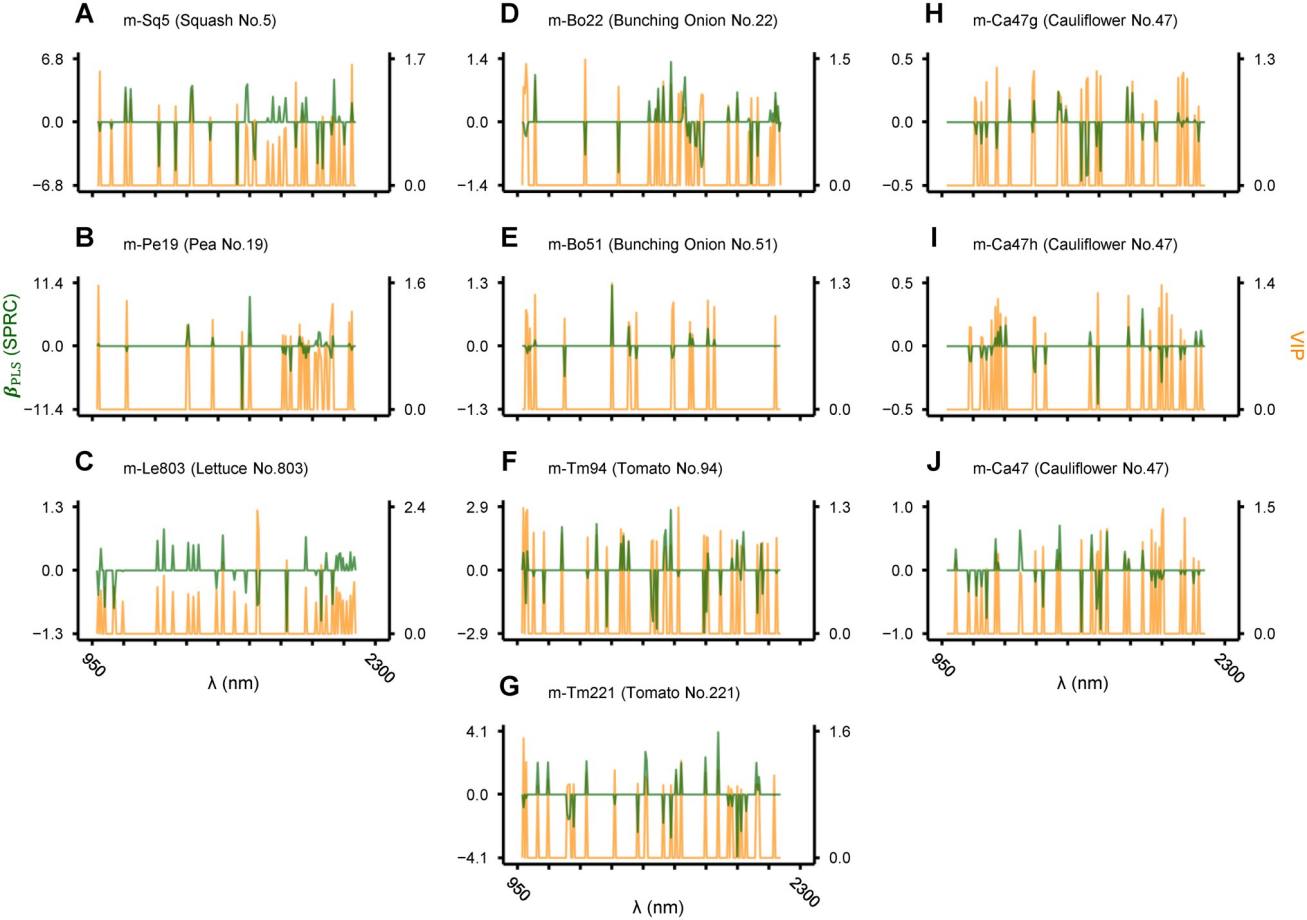
Variable importance plot of sparse discriminant models for seed quality evaluation. NIR spectrum employed as the explanatory variable and the number of wavebands selected in each model are shown in Table 4. Standard partial regression coefficients (SPRCs) and variable importance in projection (VIP) for each waveband in the indicated discriminant models are drawn in green and orange lines, respectively. The ranges of SRPC and VIP are shown on the left and right Y-axes, respectively. The range of X-axis is common to that shown at the bottom (C, G and J).

**Fig 6:**
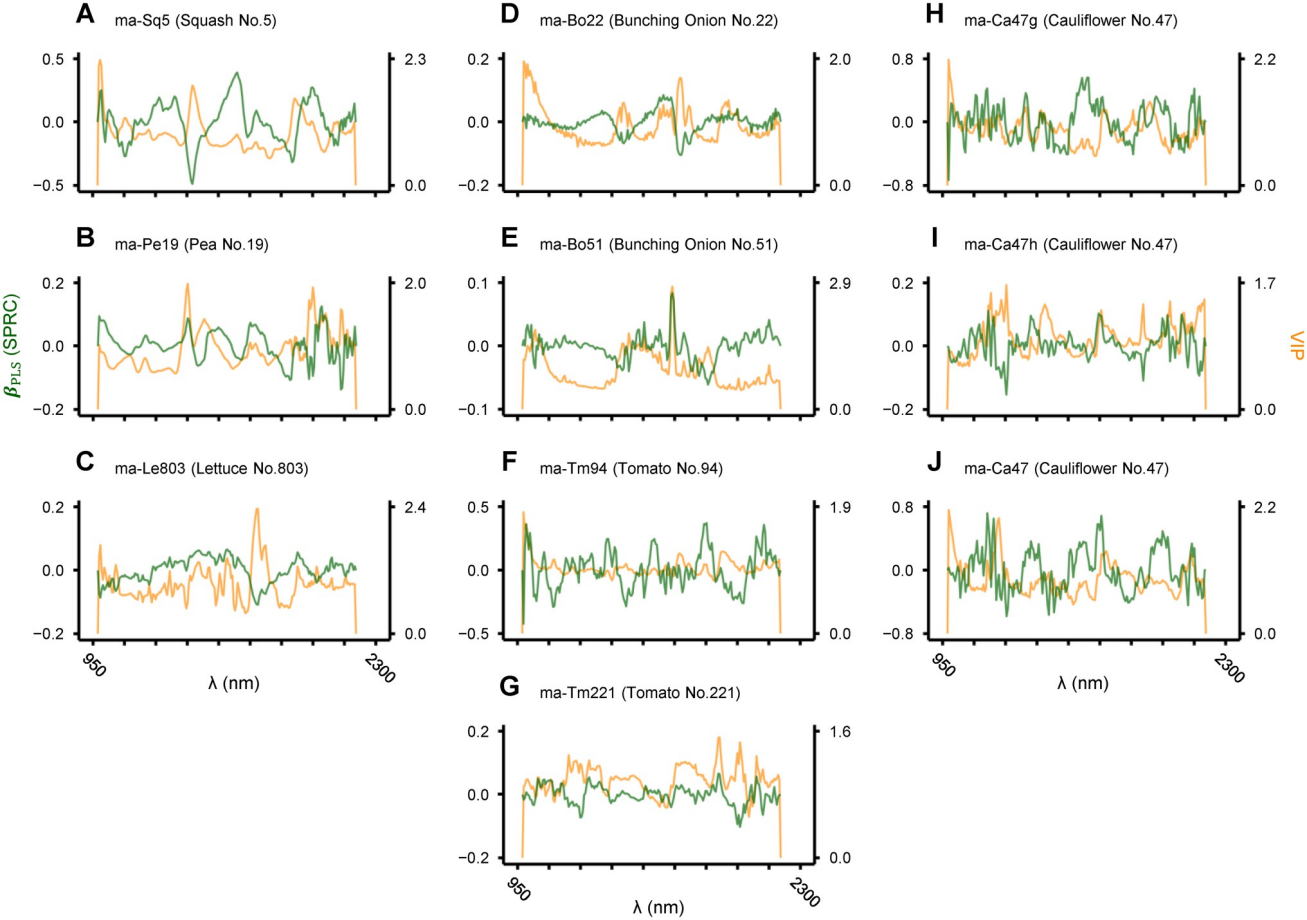
Variable importance plot of non-sparse discriminant models for seed quality evaluation. NIR spectrum employed as the explanatory variable in each model is shown in Table 4. Without variable selection, the number of wavebands incorporated in the model was 201 for all cases. The figure is drawn in the same style as Fig 5.

Figs 5 and 6 show standardized PRCs (SPRCs) and VIP for each waveband in sparse and non-sparse discriminant models derived with and without waveband selection, respectively. The magnitude of their absolute values can be interpreted as relative importance of each waveband in discrimination. As summarized in Table 4, NIR spectra (***X***_1_ to ***X***_12_) employed as the explanatory variable varied between sparse and non-sparse models, as well as between models for different crop species and cultivars. For all corresponding pairs, the number of wavebands incorporated in sparse models was 6–10 times fewer than non-sparse counterparts. Accordingly, it is easier with sparse models to identify the wavebands where the quality of seeds is reflected.

**Table 4:** Selected explanatory variables in sparse and non-sparse discriminant models for seed quality evaluation.

Target			Model											
Species	Cultivar	Quality Trait*^1^	Sparse						Non-Sparse					
			Symbol	Explanatory Variable					Symbol	Explanatory Variable				
				Spectra*^2^	Base*^3^	SG	SNV	nλ*^4^		Spectra*^2^	Base*^3^	SG	SNV	nλ*^4^
Squash	5	G	m-Sq5	***X***_6_	***R*** ^-1^	+	–	29	ma-Sq5	***X***_2_	***R***	+	–	201
Pea	19	G	m-Pe19	***X***_9_	–log***R***	–	–	27	ma-Pe19	***X***_8_	***R*** ^-1^	+	+	201
Lettuce	803	G	m-Le803	***X***_5_	***R*** ^-1^	–	–	32	ma-Le803	***X***_10_	–log***R***	+	–	201
Bunching Onion	22	G	m-Bo22	***X***_1_	***R***	–	–	35	ma-Bo22	***X***_1_	***R***	–	–	201
	51	G	m-Bo51	***X***_12_	–log***R***	+	+	16	ma-Bo51	***X***_4_	***R***	+	+	201
Tomato	94	G	m-Tm94	***X***_10_	–log***R***	+	–	33	ma-Tm94	***X***_2_	***R***	+	–	201
	221	G	m-Tm221	***X***_2_	***R***	+	–	29	ma-Tm221	***X***_4_	***R***	+	+	201
Cauliflower	47	G+H	m-Ca47	***X***_8_	***R*** ^-1^	+	+	31	ma-Ca47	***X***_2_	***R***	+	–	201
		G	m-Ca47g	***X***_12_	–log***R***	+	+	28	ma-Ca47g	***X***_2_	***R***	+	–	201
		H	m-Ca47h	***X***_12_	–log***R***	+	+	26	ma-Ca47x	***X***_4_	***R***	+	+	201

SG, Savizky-Golay; SNV, standard normal variate.

*^1^ G and H denote germinability and paternal haplotype, respectively.

*^2^ Symbols match with those in S1 Fig.

*^3^ Base spectra to which SG filtering and/or SNV transformation were applied. ***R*** denotes raw reflectance spectra.

*^4^ Number of wavebands incorporated in each model.

Table 5 compares performance of sparse and non-sparse models using metrics devised above. In all cases, sparse models outperformed their non-sparse counterparts as evaluated by *sP* and AUC-rPR. We will only focus on sparse discriminant models hereafter.

**Table 5:** Performance of sparse and non-sparse discriminant models in internal validation compared on the same dataset.

Target			Dataset		Model													
					Sparse							Non-Sparse						
Species	Cultivar	Quality Trait	Symbol	*iP*	Symbol	Performance						Symbol	Performance					
						Standard			Overall				Standard			Overall		
						sLST	*sP* (= *sR*)	*sA*	*A*max	AUC-PR	AUC-rPR		sLST	*sP* (= *sR*)	*sA*	*A*max	AUC-PR	AUC-rPR
Squash	5	G	d-Sq5A1	80.8%	m-Sq5	0.2665	92.8%	88.3%	89.6%	0.9726	0.8571	ma-Sq5	0.4243	89.2%	82.5%	87.9%	0.9502	0.7402
Pea	19	G	d-Pe19A1	64.9%	m-Pe19	0.0677	83.0%	77.9%	77.9%	0.8864	0.6761	ma-Pe19	0.2109	81.9%	76.4%	77.2%	0.8456	0.5599
Lettuce	803	G	d-Le803A1	83.9%	m-Le803	0.3276	89.6%	82.5%	87.1%	0.9440	0.6511	ma-Le803	0.4797	87.5%	79.1%	84.9%	0.9193	0.4974
Bunching Onion	22	G	d-Bo22A1	75.8%	m-Bo22	0.2603	85.4%	77.9%	80.0%	0.8909	0.5494	ma-Bo22	0.2989	84.6%	76.6%	81.2%	0.8722	0.4796
	51	G	d-Bo51A1	81.3%	m-Bo51	0.2968	86.9%	78.8%	82.1%	0.8860	0.3993	ma-Bo51	0.3983	85.6%	76.7%	82.5%	0.8739	0.3273
Tomato	94	G	d-Tm94A1	75.6%	m-Tm94	0.1966	92.9%	89.3%	90.2%	0.9659	0.8604	ma-Tm94	0.2724	90.2%	85.1%	87.2%	0.9323	0.7227
	221	G	d-Tm221A1	47.3%	m-Tm221	0.0018	84.7%	85.5%	86.7%	0.9152	0.8391	ma-Tm221	0.0418	83.4%	84.3%	84.6%	0.8975	0.8056
Cauliflower	47	G+H	d-Ca47A1	69.7%	m-Ca47	0.1817	86.6%	81.4%	82.6%	0.9381	0.7958	ma-Ca47	0.2259	84.2%	78.0%	79.2%	0.9163	0.7236
		G	d-Ca47gA1	87.9%	m-Ca47g	0.4059	94.9%	91.1%	92.8%	0.9814	0.8460	ma-Ca47g	0.3971	94.2%	89.8%	91.1%	0.9716	0.7647
		H	d-Ca47xA1	79.4%	m-Ca47h	0.2592	90.0%	84.2%	85.1%	0.9620	0.8155	ma-Ca47x	0.2163	88.3%	81.4%	82.8%	0.9408	0.7123

Abbreviations not mentioned are common to those in Tables 3 and 4.

#### (2) Distribution of discriminant scores

**Fig 7:**
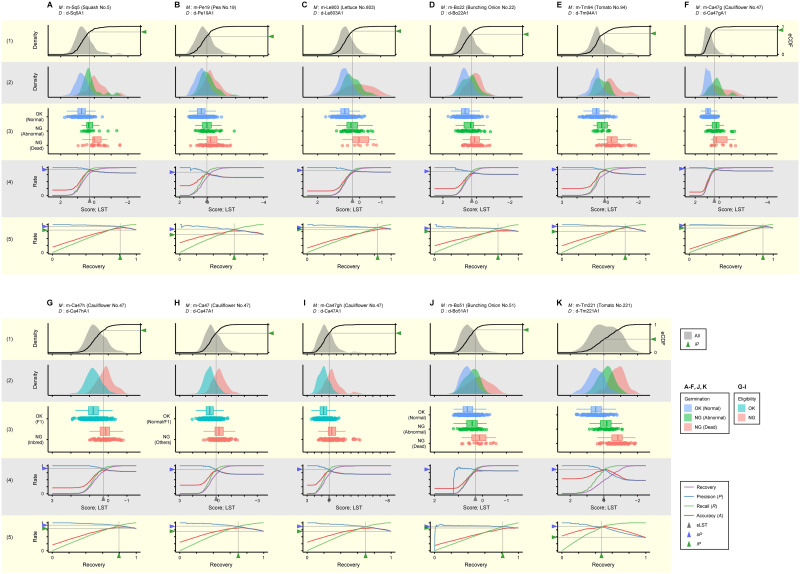
Internal validation of discriminant models for seed quality evaluation. The figure illustrates the results of internal validation with real discriminant model *M* applied to dataset *D* in the same style as Fig 3. Summary of datasets, discriminant models, and the performance of discrimination with each (*M*, *D*) combination are shown in Tables 2, 4 and 5, respectively. In the cases of predicting germinability of seeds (A–K, J, K), score distribution of ineligible seeds is drawn separately for abnormally germinated and non-germinated seeds.

As evident in Table 5, all selected models worked as expected in that *sP* exceeded *iP*, albeit with varying extent. Fig 7 shows distribution of discriminant scores given by each model to individual seeds (rows 1–3) and performance metrics in relation to LST (row 4) or recovery rate of seeds (row 5) as in Fig 3. For most models, the graphs were similar to Fig 3B drawn by the hypothetical model MP, suggesting that they have practical, if not perfect, discrimination performance. By contrast, those for the model m-Bo51 derived for bunching onion cultivar No.51 were more like Fig 3C drawn by the PP model. The inferior performance of this model is also evidenced by the low relative value of AUC-rPR (0.3993; *see* Table 5).

Out of 10 pairs of models listed in Tables 5 and 8 are intended for predicting germinability of seeds. In deriving these models, both non-germinated and abnormally germinated seeds were equally labeled as ineligible. Nevertheless, seeds normally germinated occupied the highest score range, followed by abnormally germinated and then non-germinated seeds in all cases regardless of target species and cultivars (Fig 7A–7F, 7J and 7K; rows 2 and 3). Thus, scores given by these models may reflect not only the probability of normal germination but also the inherent vigor of seeds.

#### (3) Application domain of discriminant models

The performance evaluation of discriminant models in Table 5 and Fig 7 is based on outer cross-validation set to avoid overestimation, but is not more than internal validation using data from the seeds with which the models were trained. To better estimate the generalization performance and application domain of each model, seeds from (1) different batch of the same lot, (2) different lot of the same cultivar, and (3) different cultivar of the same species, relative to those used for model training, were applied to external validation. The results are shown in Table 6 and S2 Fig, which are arranged in correspondence with Table 5 and Fig 7, respectively.

**Table 6:** External validation of discriminant models for seed quality evaluation.

Target			Dataset		Model						
Species	Cultivar	Quality Trait	Symbol	*iP*	Symbol	Performance					
						Standard			Overall		
						sLST	*sP* (= *sR*)	*sA*	*A*max	AUC-PR	AUC-rPR
Squash	5	G	d-Sq5A2	69.6%	m-Sq5	0.4962	82.4%	75.5%	78.0%	0.9079	0.6973
Pea	19	G	d-Pe19A2	49.8%	m-Pe19	-1.0225	85.6%	85.6%	85.8%	0.8828	0.7664
Lettuce	803	G	d-Le803A2	86.3%	m-Le803	0.4333	88.4%	80.1%	86.3%	0.9200	0.4176
Bunching Onion	22	G	d-Bo22A2	66.1%	m-Bo22	0.0538	83.8%	78.6%	80.2%	0.8884	0.6711
			d-Bo51A1	81.3%		0.3861	82.8%	72.1%	81.6%	0.8281	0.0864
	51		d-Bo22A1	75.8%	m-Bo51	0.6701	79.9%	69.5%	77.0%	0.8115	0.2216
Tomato	94	G	d-Tm94B1	81.0%	m-Tm94	0.4120	90.8%	85.1%	88.7%	0.9295	0.6298
			d-Tm221A1	47.3%		0.5577	77.4%	78.6%	80.4%	0.8297	0.6770
	221	G	d-Tm94A1	75.6%	m-Tm221	0.4824	85.8%	78.6%	81.4%	0.9007	0.6011
Cauliflower	47	G+H	d-Ca47A2	66.7%	m-Ca47	0.2537	81.7%	75.6%	77.0%	0.8883	0.6648
			d-Ca47B1	62.6%		0.1549	80.6%	75.6%	75.8%	0.8600	0.6254
		G	d-Ca47gA2	86.2%	m-Ca47g	0.0404	93.4%	88.7%	90.5%	0.9654	0.7487
			d-Ca47gB1	75.6%		-0.0424	92.5%	88.6%	88.9%	0.9579	0.8270
		H	d-Ca47hA2	77.7%	m-Ca47h	0.2253	86.7%	79.4%	81.3%	0.9342	0.7051
			d-Ca47hB1	84.3%		-0.0913	90.8%	84.6%	86.2%	0.9785	0.8626

Abbreviations not mentioned are common to those in Tables 3 and 4.

As usual in discriminant analysis, the models performed worse in most cases in external than internal validation (Tables 5 and 6). Nevertheless, insofar as the dataset was from the cultivar the model was intended for, the deterioration in discrimination performance was not that severe as to impair its practicality. This is easier to grasp by comparing AUC-rPR.

**Fig 8:**
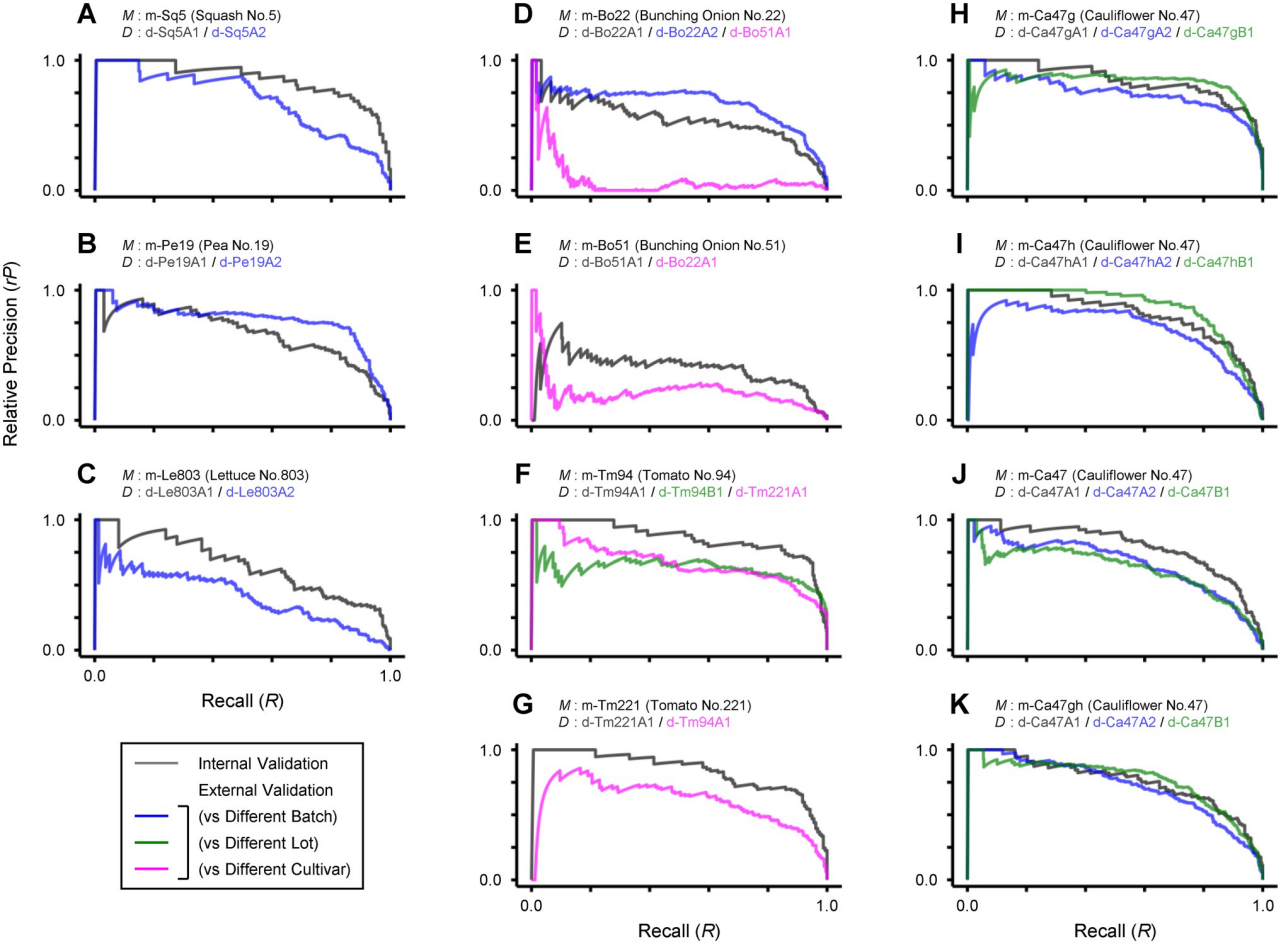
Relative precision-recall (rPR) curves in eligible seed sorting by discriminant models derived for various crop cultivars. Shown are rPR curves for discrimination with model *M* applied to dataset *D*. Summary of datasets, discriminant models, and the performance of discrimination with each (*M*, *D*) combination in internal and external validations, respectively, are shown in Tables 2 and 4–6. Different line colors are used according to the type of validation (dark gray for internal validation) and, for the cases of external validation, whether the dataset under analysis is from different batch (blue), lot (green), or cultivar (magenta) compared to the internal dataset for model *M*. The range of X-axis is common to that shown at the bottom (C, G and K).

S3 Fig and Fig 8 show PR and rPR curves, respectively, in discrimination with all model and dataset combinations, including internal and external validations. Again, most curves, at least for internal validation, were similar to Fig 4B drawn by the MP model, while those for m-Bo51 more like Fig 4C by the PP model. Between different cultivars of the same species, the model m-Tm94 derived for tomato cultivar No. 94 performed reasonably on a dataset from the cultivar No. 221 (d-Tm221A1) and *vice versa* (m-Tm221 versus d-Tm94A1) (Fig 8E amd 8F). By contrast, the model m-Bo22 derived for bunching onion cultivar No.22 performed even worse than m-Bo51 on a dataset from the cultivar No.51 (d-Bo51A1) (Fig 8D and 8E).

#### (4) Consistency of discriminant scores

While discriminant scores of eligible seeds were always distributed on a higher range than those of ineligible seeds, the absolute scores deviated for each testing occasion, even in cases where seed batches from the same lot were used. This is evident from the discrepancy of sLST between internal and external validations, and, if any, between multiple external validations (Tables 5 and 6), or can be noticed by comparing the corresponding sets of graphs in Fig 7 and S2 Fig.

Since NIR hyperspectral images of seeds, the source of discriminant scores, were taken at each testing occasion, changes in the condition of seed materials or the imaging system over time could have compromised the consistency of absolute scores. Such state changes cannot be completely prevented in practice, so the validity of discriminant scores like sLST is ad hoc. As mentioned, however, LST is convertible to seed recovery rate, and similarly, absolute discriminant scores to relative quality rank of seeds. Thus, the adjustment of discrimination strength should be done by setting LST that back-calculated from the target seed recovery rate each time, not LST that worked well in the past.

### Quality evaluation of cauliflower seeds for commercial value

As mentioned, each seed of cauliflower cultivar No. 47 is regarded as fully eligible, i.e., commercially valuable, only if it is F1 hybrid and can germinate normally. Whether these two requirements are met can be predicted either separately or together. Then which works better in terms of performance and usability in discriminating fully eligible cauliflower seeds? Below, we compare methods for seed quality evaluation involving multiple trait requirements.

#### (1) Evaluation by successive per-trait discrimination

Among discriminant models derived above, the model m-Ca47g predicts whether a cauliflower seed can germinate normally regardless of its paternal haplotype. The model m-Ca47h, on the other hand, was derived to predict only whether a cauliflower seed is an F1 hybrid, while ignoring its germinability. Unlike other models, these two need to be used in combination to discriminate whether a seed of interest is fully eligible.

**Fig 9:**
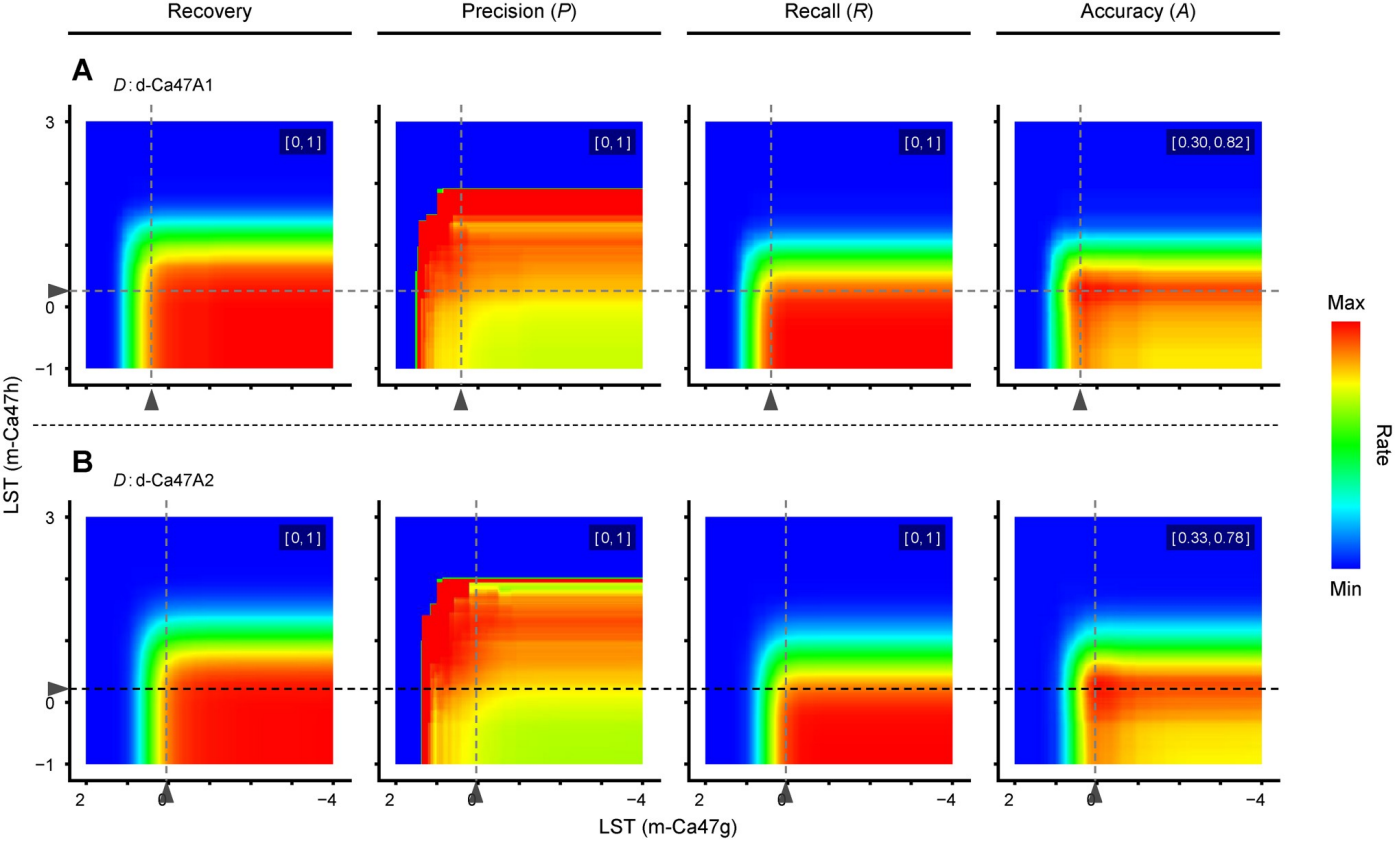
Performance of quality sorting for fully eligible cauliflower seeds by successive per-trait discrimination. Shown are the results of internal (A) and external (B) validations. The value range for the color scale is indicated at the top right of each plot. The X- and Y-axes represent lower score threshold (LST) for discrimination with the models m-Ca47g and m-Ca47h, respectively Gray arrowheads indicate standard LST (sLST) for the respective model when applied to the indicated dataset *D*. The range of both axes are common to all plots.

In case such “successive” method is to be taken, the relationship between LST settings for the two models and the metrics related to discrimination performance for fully eligible seeds becomes three-dimensional. This can be represented planarly using heatmaps as in Fig 9. PR and rPR relationships will also be three-dimensional, i.e., curved surfaces instead of curved lines, as depicted in S4 Fig. Optimizing the discrimination strength is equivalent to finding coordinates on Fig 9 where *P* is above the desired level and *R* within an acceptable range. The fact is that, however, the relationships as in Fig 9 are yet black boxes when a discriminant model is first applied to a novel seed batch for which the individual eligibility has not been clarified. The same is true for S2 Fig; at this stage, distribution of scores can be estimated for all seeds as in row 1, but not by class as in rows 2–3.

Table 7 shows the volume under PR and rPR curved surfaces (denoted as “AUC-PR and rPR” for consistency), along with performance metrics when both m-Ca47g and m-Ca47h models are used under their respective standard condition. While AUC-rPR is sufficiently high, a severe disadvantage of this method is the difficulty in adjusting discrimination strength to achieve desired *P* in case *sP* fell short of it.

**Table 7:** Performance comparison of different methods for discriminating fully eligible cauliflower seeds.

Method	Validation Type	Dataset		Model							
		Symbol	*iP*	Symbol	Performance						
					Standard				Overall		
					sLST		*sP* (= *sR*)	*sA*	*A*max	AUC-PR	AUC-rPR
Successive	Internal	d-Ca47A1	69.7%	m-Ca47g + m-Ca47h	0.4059	*	85.8%	79.4%	82.0%	0.9315	0.8212
					0.2592						
	External	d-Ca47A2	66.7%		0.0404	*	81.6%	76.4%	77.7%	0.9331	0.8093
					0.2253						
		d-Ca47B1	62.6%		-0.0424	*	82.9%	78.6%	80.5%	0.9165	0.8119
					-0.0913						
Direct	Internal	d-Ca47A1	69.7%	m-Ca47	0.1817		86.6%	81.4%	82.6%	0.9381	0.7958
	External	d-Ca47A2	66.7%		0.2537		81.7%	75.6%	77.0%	0.8883	0.6648
		d-Ca47B1	62.6%		0.1549		80.6%	75.6%	75.8%	0.8600	0.6254
Unification	Internal	d-Ca47A1	69.7%	m-Ca47gh	-0.0364		85.1%	79.2%	82.0%	0.9271	0.7593
	External	d-Ca47A2	66.7%		0.0321		83.0%	77.3%	77.4%	0.9081	0.7244
		d-Ca47B1	62.6%		0.0043		82.9%	78.6%	80.2%	0.9114	0.7628

Abbreviations not mentioned are common to those in Table 3.

* Upper and lower denote the values for m-Ca47g and m-Ca47h models, respectively.

#### (2) Direct evaluation omitting per-trait discrimination

In evaluating the commercial value of seeds, it is not necessarily required to discriminate their quality per-trait. In contrast to the precedent, the model m-Ca47 only predicts whether a cauliflower seed is fully eligible, regardless of whether its germinability or paternal haplotype is ineligible. The properties of this model have already been presented alongside those of other models (Figs 7H and 8J and S2H and S3J Figs).

As summarized in Table 7, metrics related to standard performance were comparable between cases when the “direct” method with the model m-Ca47 or the “successive” method referring to both m-Ca47g and m-Ca47h scores was taken. As seen in Fig 7H and S2H Fig, m-Ca47 has the advantage that the discrimination strength can be adjusted through a single LST, while the performance is practically high. Another advantage of the “direct” method is only one discriminant model is sufficient to be derived regardless of the number of quality traits that should be focused on. This method can be taken if its disadvantage in unpredictability of per-trait quality of seeds, i.e., germinability and haplotype, is acceptable.

#### (3) Evaluation by unified score of multiple per-trait discrimination

To parallelize the advantages of “successive” and “direct” methods as described above, i.e., predictability of per-trait quality and high adjustability of discrimination strength, we explored how to integrate scores from multiple discriminant models into a single representative score.

As mentioned, distribution of discriminant scores can vary between testing occasion even those given by a single model to seed batches from the same lot. Also, the scores given by different discriminant models are not comparable with each other. To compensate for such inconsistent nature of primary discriminant scores, we propose the following score conversion analogous to variable standardization:

$$\hat{z}\left(M,\;D\right)\equiv \frac{\hat{y}\left(M\right)-\text{sLST}\left({\hat{y}}_{p}\left(M,\;D\right)\right)}{s\left({\hat{y}}_{p}\left(M,\;D\right)\right)}$$ (2)

where (*M*, *D*) denotes combined use of the model *M* and dataset *D* (usually devoid of ***y***), and ***ŷ*** denotes the primary scores. ***ŷ***_***p***_ is a subset of ***ŷ*** for predicting the standard deviation (SD) and sLST of ***ŷ***, with the respective estimates denoted as *s*(***ŷ***_***p***_) and sLST(***ŷ***_***p***_).

In evaluating seed quality based on multiple traits, it is reasonable to take the lowest rating as the representative, as rejection should be preferred over acceptance in case of doubt. The nature of ***ẑ*** as being “standardized” merits in unifying scores from multiple discriminant models. When the quality of seeds in dataset *D* is evaluated using *n* models, *M*_1_ to *M*_n_, the unified discriminant score can be represented as:

$$\hat{u}\left(D\right)\equiv \underset{1\leq i\leq n}{\text{min}}\;\left\{\hat{z}\left(M_{i},D\right)\right\}$$ (3)

The score ***û*** can again be treated alike the score ***ŷ***, and sLST for this can also be determined.

The m-Ca47gh is an integrated model of m-Ca47g and m-Ca47h, in which the primary ***ŷ*** scores from the respective source models are unified through the above procedures. The properties of m-Ca47gh in internal and external validations are shown in Fig 7I and S2I Fig, and PR and rPR curves for discrimination by this model in S3K Fig and Fig 8K, respectively. According to rPR curves (Fig 8J and 8K) and AUC-rPR in Table 7, performance deterioration in external compared to internal validation, a measure of model overfitting, was less pronounced with the model m-Ca47gh than m-Ca47. This “unification” method can be taken if a set of discriminant models with reasonable performance is available, each for predicting one of multiple quality traits.

### Visualization of discriminant scores and verification of seed quality

**Fig 10:**
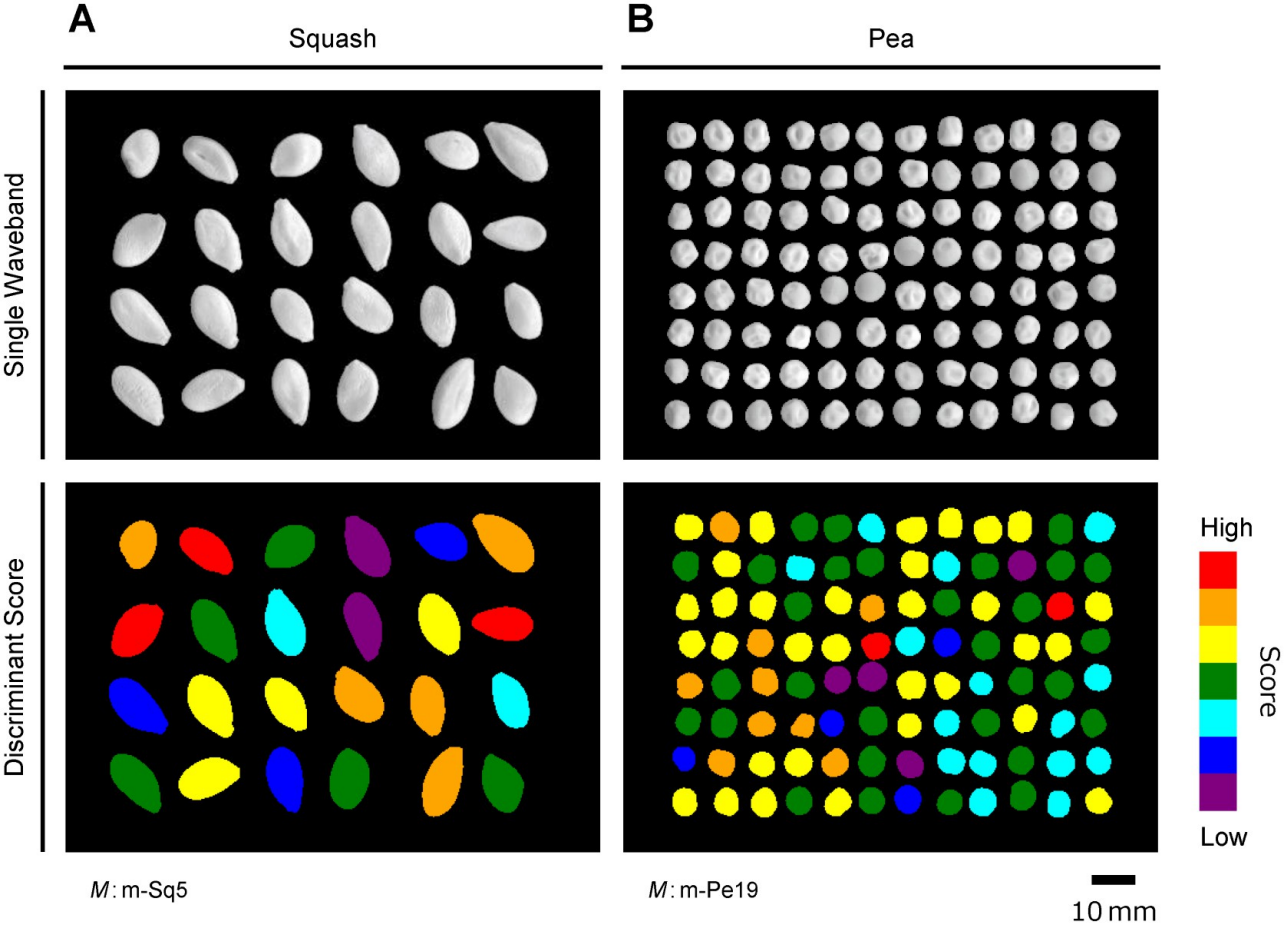
Visualization of predicted quality rank of crop seeds by direct processing of NIR hyperspectral images. Each seed of squash (A) and pea (B) were scored by discriminant models (*M*) m-Sq5 and m-Pe19, respectively. Summary of the models are shown in Table 4. Seeds within the same field-of-view (FOV) are filled with a pseudo-color according to the relative score of each seed. The figure can be reproduced using the software and data files provided as S2 File.

To verify the feasibility of automatic eligible seed sorting based on the discriminant models derived above, we developed a system to directly visualize the discriminant scores of seeds within the FOV of NIR hyperspectral images. Fig 10 shows the quality of squash and pea seeds as predicted using the models m-Sq5 and m-Pe19, respectively. The program and data files for retesting are provided as S2 File. The same is possible for seeds from crop species other than squash and pea.

**Fig 11:**
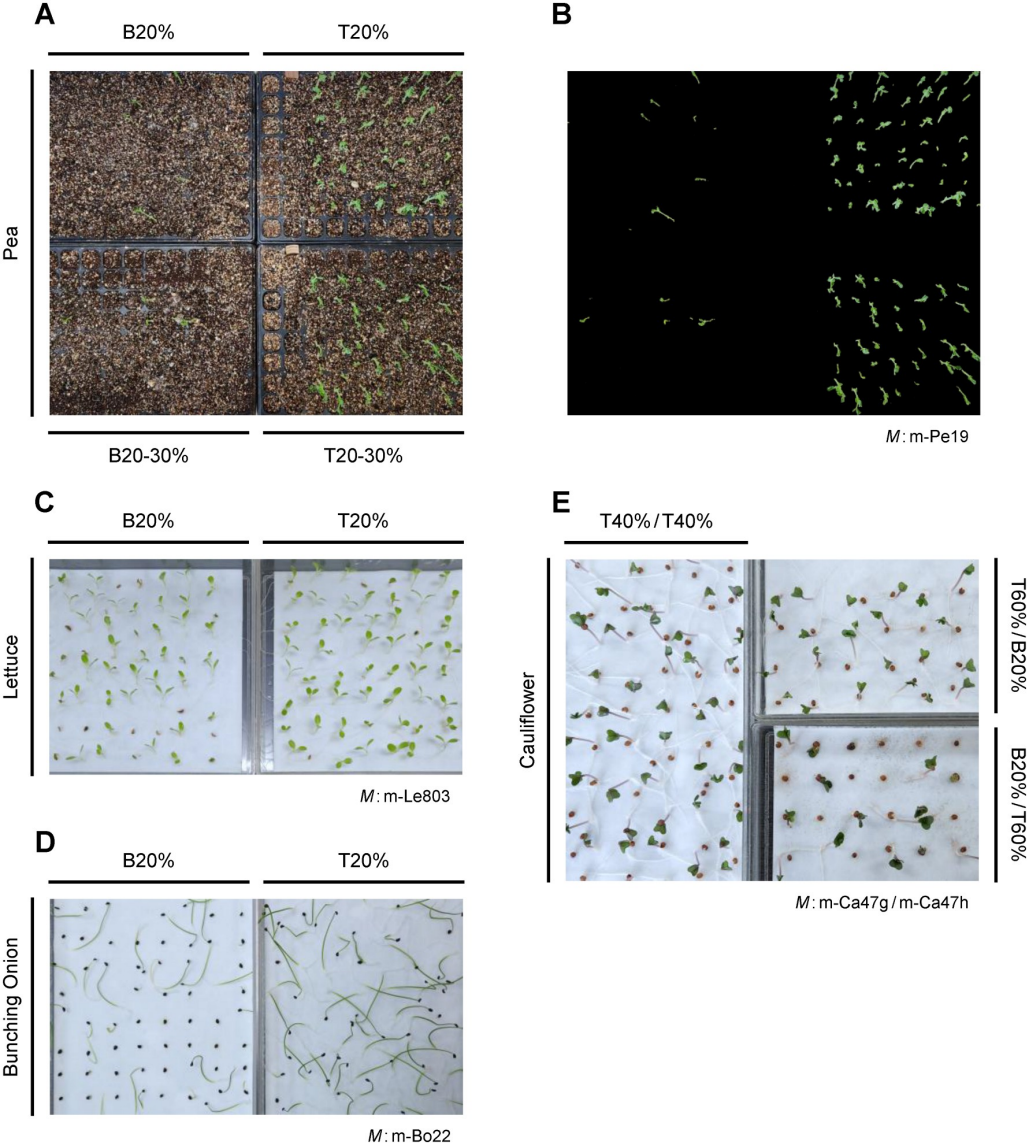
Germination and post-emergence growth of crop seeds with different predicted quality ranks. The test results are summarized in Table 8. Seeds were scored by the discriminant model (*M*) intended for each crop cultivar and classified into rankers from top (T) to bottom (B). They were then sown on germination beds at the same time. The images were taken before the test deadline. For peas, the original image (A) along with the extracted image of seedlings (B) are shown. Cauliflower seeds in (E) were scored by two discriminant models m-Ca47g and m-Ca47h, and ranked for each quality trait of germinability and paternal haplotype.

We then separated high- and low-ranked seeds on the basis of score images and tested for their germinability and/or paternal haplotype. Table 8 summarizes the results of cultivation tests where seeds of different predicted quality ranks were sown simultaneously. Fig 11 shows germination beds photographed before the deadline. For all crop species, normal germination was observed more frequently from batches of high-ranked than of low-ranked seeds. For cauliflower cultivar No. 47, more inbred seedlings emerged from batches of seeds with lower m-Ca47h scores (Table 8).

**Table 8:** Seed quality breakdown by rank as predicted by discriminant models.

Species	Dataset		Model	Score Rank*^1^		Quality Breakdown									
	Symbol	*iP*	Symbol			Germination (G)			Haplotype (H)			Eligibility (G+H)		Total	Precision (*P*)*^3^
						Normal	Abnormal	Dead	F1	Inbred	ND*^2^	OK	NG		
Pea	d-Pe19A2	49.8%	m-Pe19	T20%		76	4	4	–	–	–	76	8	84	90.4%
				T20-30%		59	6	6	–	–	–	59	12	71	83.0%
				B20-30%		10	9	41	–	–	–	10	50	60	16.7%
				B20%		11	10	80	–	–	–	11	90	101	10.9%
Bunching Onion	d-Bo22A2	66.1%	m-Bo22	T20%		134	10	3	–	–	–	134	13	147	91.2%
				B20%		79	53	79	–	–	–	79	132	211	37.4%
Lettuce	d-Le803A2	86.3%	m-Le803	T20%		173	7	1	–	–	–	173	8	181	95.6%
				B20%		118	26	15	–	–	–	118	41	159	74.2%
Cauliflower	d-Ca47B1	62.6%	m-Ca47g + m-Ca47h	T40%	*^4^	107	4	0	109	0	2	106	5	111	95.5%
				T40%											
				T60%	*^4^	72	1	0	38	34	2	38	36	74	51.4%
				B20%											
				B20%	*^4^	16	46	20	49	1	32	15	67	82	18.3%
				T60%											

Abbreviations not mentioned are common to those in Table 3.

*^1^ T and B denote top and bottom, respectively.

*^2^ Not determined because tissue extracts could not be prepared from dead or abnormally-germinated seeds.

*^3^ Synonymous to eligibility rate after sorting.

*^4^ Upper and lower denote ranks predicted by m-Ca47g and m-Ca47h models, respectively, for germinability and paternal haplotype.

As concerned from the low relative value of AUC-rPR (0.4176, *see* Table 6), the efficacy of seed sorting in lettuce was inferior to that in other crop species listed in Table 8. Nevertheless, growth of lettuce seedlings from high-ranked seeds was apparently more vigorous than those from low-ranked seeds.

## Discussion

We demonstrated a method for seed quality evaluation widely applicable to a variety of crop species, which is based on NIR imaging spectrometry and machine learning techniques. In most cases presented here, seed batches with higher discriminant scores were more enriched in eligible seeds (Table 8 and Fig 11). On the other hand, we also encountered cultivars for which no satisfactory models for discriminating eligible seeds could be derived (e.g., bunching onion cultivar No. 51; *see* Table 5 and Fig 8E). Including such unfortunate cases, the advantage of the method is that it takes only a couple of hours from the completion of routine seed quality inspections, such as germination tests, until it becomes clear whether discrimination of eligible seeds is possible for the given seed lot. Where possible, the derived discriminant model can be implemented in a sorting device to immediately start “repairing” the seed lots, that otherwise do not meet quality standards and have no choice but to be discarded. The only operation required more than usual is to capture a NIR hyperspectral image of seeds before subjecting them to quality inspection; this allows to establish a dataset linking their spectroscopic and quality traits as the explanatory and objective variables, respectively (S1 Fig, step 1).

When aiming for pass/fail discrimination by machine learning techniques, we tend to expect a discriminant model once derived being usable semi-permanently for the same purpose. However, at least in our experience, the crop species and cultivars that produce ineligible seed lots are not necessarily fixed; the occurrence of such lots often is accidental and becomes the very first opportunity for troubleshooting in that troubled cultivar. In the case of cauliflower seeds, differences in physicochemical properties between F1 hybrid and inbred should be consistent regardless of seed lot. By contrast, there are various causes for the occurrence of poorly germinating seeds, and hence it makes sense to derive an optimal model for discriminating eligible seeds on a case-by-case basis. For example, the properties of seeds that have lost the potential to germinate normally due to high temperature or over-humidity are different, and therefore the boundaries (possibly defined as hyperplanes or hypersurfaces) separating them from eligible seeds should also be different from each other. This seems consistent with the observation that combined use of the models (m-Ca47g and m-Ca47h) specialized for predicting germinability or paternal haplotype was somewhat better in overall performance than using a single model (m-Ca47) when discriminating fully eligible cauliflower seeds (Table 8). Even eligible seeds themselves, unlike industrial products, can have somewhat different properties from lot to lot, so that it would also be difficult to define an enclosed boundary separating them from ineligible seeds of any kinds.

Although we mentioned it takes only a short while, the procedure we employed to derive discriminant models for seed quality evaluation is not necessarily computationally efficient (S1 Fig, steps 2–4; S1 File). To promptly respond to the case-by-case needs for seed quality reparation, efficient and rapid procedures should be explored continually. Sparse PLS and its variants [22, 23] are the ones we should try early for deriving discriminant models with high interpretability and performance; these may help reduce or even eliminate the need for iterative model derivation due to their high reproducibility. Whatever methods used for deriving discriminant models though, the concepts and metrics presented in this study, such as “standard” condition and performance as well as “AUC-rPR”, will help systematically proceed with model selection.

One of the issues not fully addressed in this study is exploring the implications of the derived discriminant models (Fig 5). Near to mid-wave infrared (IR) light induces vibrational level changes in wavelength-specific chemical bonds, and hence the IR spectrum of a substance acts like a fingerprint of its chemical composition. Additionally, IR light to the shortwave range is suitable for non-destructive measurement [6]. Based on an observation that coniferous tree species for afforestation (Cupressaceae and Pinaceae) tend to form sterile seeds with abnormality in endosperm storage lipids, we have developed and put into practice a method and device for isolating fertile seeds in these species. There we focused on the property of methylene group (-CH_2_-), that is abundant in fatty acid side chains and preferentially absorbs NIR lights centered at 1,730 nm (1st overtone of antisymmetric stretching) or 1,200 nm (2nd overtone of symmetric stretching) [24, 25]. Conversely, in an attempt to estimate the elemental composition of seeds (in rice and cypress), we noticed that absorption wavebands of the peptide group (-CONH-) were preferentially selected in sparse linear models for regressing nitrogen content (e.g., around 1,920 nm attributable to 2nd overtone of amide I (C = O) stretching). Peptide groups are abundant in proteins, the major storage forms of nitrogen in seeds. In this way, dissection of the wavebands selected in sparse discriminant models and the magnitude of SPRC and/or VIP for each waveband as in Fig 5 helps to clarify the substances that make the difference between eligible and ineligible seeds. Accumulation of such information will be a precious knowledge base for realizing a management system to stably maintain and supply high-quality seeds.

Another technical issue that remains to be solved is how to spot an appropriate LST to achieve *P* (eligibility rate after sorting) nearly in line with expectations. Though *sP* (*P* under standard condition) is somewhat affected by *iP* (eligibility rate before sorting), it roughly averaged 86% when discriminant models were externally validated on seeds batches from the cultivars they were intended for (Table 6). This rate is likely unsatisfactory for most seedling producers, so LST must be raised at the cost of some decline in *R* (recovery rate of eligible seeds), i.e., more of eligible seeds to be discarded. The relationship between *P* and recovery rate of seeds usually have a convex shape as in Fig 3B (row 5; *see* also Fig 7 and S2 Fig) unless using a discriminant model with poor performance as in Fig 3C (e.g., *see* Fig 7J). Hence, this may be approximated at worst as a straight line passing through the points (0, 1) and (1, *iP*). However, while making efforts on verifying the feasibility of seed quality evaluation in as many crop species and cultivars as possible, we could not spare much effort on replicate tests using as many seed batches from the same cultivar as possible. We are not yet at the stage we can propose a reliable means to adjust the discrimination strength as intended.

Ever since the technology of seed quality evaluation using NIR imaging spectrometry began to emerge in the mid-2000s, the high cost of the equipment required has been pointed out as an impediment to its widespread adoption. What we think is yet another impediment is that there is a limit to the number of seeds that can be handled by manual or semi-mechanical operations, whether for industrial applications or basic research. In fact, the task of capturing hyperspectral images itself is not that hard at all. Instead, what is arduous is preparing the aforementioned datasets in which the spectroscopic and quality traits of individual seeds are linked one-to-one; they all need to be processed consistently in strict order. Realizing a mechanical device that can freely manipulate (align and separate) lightweight, irregular-shaped seeds is a real challenge that requires interdisciplinary efforts. However, the time has come to confront and overcome this challenge if we are to further deepen the technology and promote its social implementation.

Single-kernel sorters equipped with NIR point spectrometers have been commercialized, and industrial use of models that achieved high extreme throughput is progressing [26, 27]. However, seeds are not homogeneous and so the trait at a single point within a seed do not necessarily represent the trait of the whole seed. Compared to spectrometers, hyperspectral cameras require a longer time for a single exposure, but have an advantage in that they can acquire spatial and spectral information of a large number of seeds at once, providing a chance to accurately estimate the biological traits of individual seeds. As presented in this study, NIR hyperspectral images allow seed quality evaluation in a wide variety of crop species and cultivars according to the same principles and procedures. We are working toward early realization of a single-kernel sorter equipped with a NIR hyperspectral camera, which will pave the way to solve the problem of “seed loss and waste” in crop production.

## Supporting information

S1 Fig Procedures of deriving discriminant models for seed quality evaluation. Details are given in Materials and Methods section. The procedures can be retested using S1 File on the R statistical software. MSE, mean squared error.
(TIF)

S2 Fig External validation of discriminant models for seed quality evaluation. The figure is drawn in the same style as Fig 7, except that the models for bunching onion cultivar No.51 and tomato cultivar number No.221 present in Fig 7J and 7K were not validated for external datasets.
(TIF)

S3 Fig Precision-recall (PR) curves in eligible seed sorting by discriminant models for various crop cultivars. The figure is drawn in the same style as Fig 8. The vertical axis indicates precision (*P*) in place of relative precision in Fig 8.
(TIF)

S4 Fig Precision- and relative precision-recall relationships in discrimination for fully eligible cauliflower seeds by “successive method”. Curves (curved surfaces) representing precision-recall (PR) (A) and relative precision-recall (rPR) (B) relationships are drawn planarly in head maps. X- and Y-axes represent recall (*R*) in per-trait discrimination with m-Ca47g and m-Ca47h models, respectively. Color scales represent closed intervals of [0.6, 1.0] in (A) and [0, 1] in (B). Initial precision (*iP*) of the dataset *D* under analysis are indicated on upper right of each heat map in (A). Results for the dataset d-Ca47A1 are of internal, and the others of external validations.
(TIF)

S1 File Scripts and data files for retesting the procedures in S1 Fig. (ZIP)

S2 File Program and data files for reproducing Fig 10. (ZIP)
