# Supplementary figures and images for: Last-percent improvement in eligibility rates of crop seeds based on quality evaluation using near-infrared imaging spectrometry
Source: PLoS One. 2023 Sep 20;18(9):e0291105. doi: 10.1371/journal.pone.0291105 (PMC10511137; doi:10.1371/journal.pone.0291105)

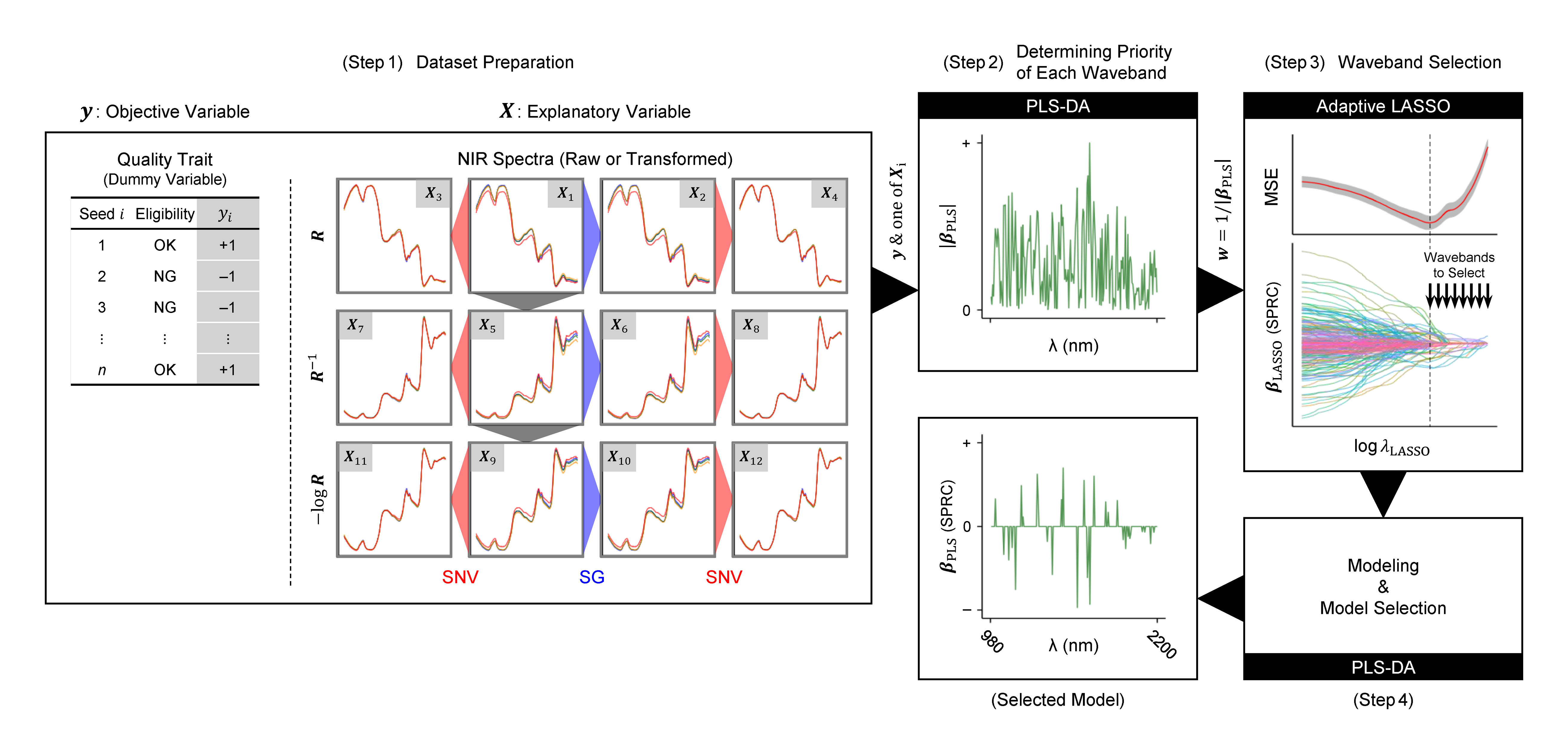

Supplement: S1 Fig — Details are given in Materials and Methods section. The procedures can be retested using S1 File on the R statistical software. MSE, mean squared error. (TIF) [file pone.0291105.s001.tif]

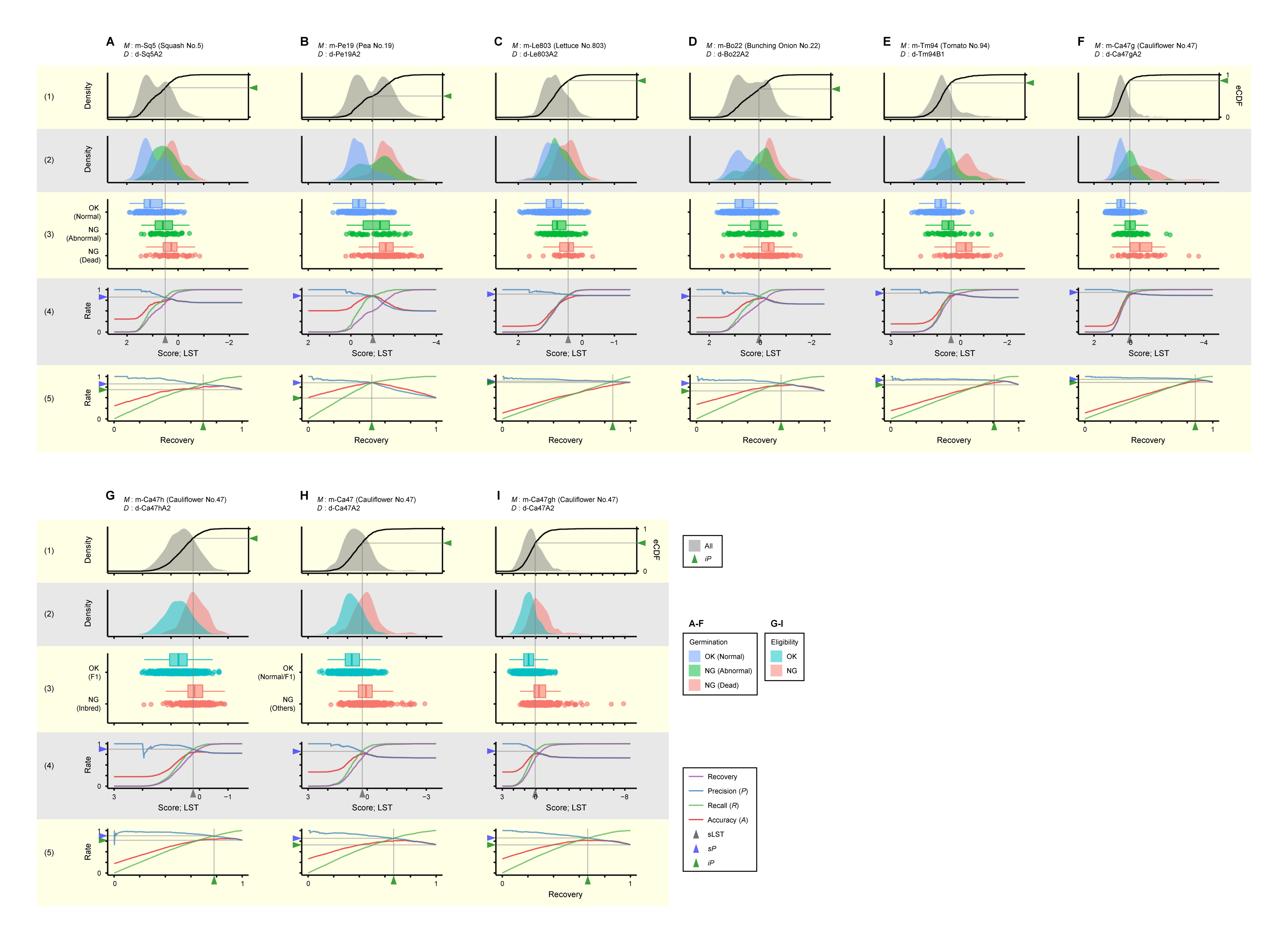

Supplement: S2 Fig — The figure is drawn in the same style as Fig 7, except that the models for bunching onion cultivar No.51 and tomato cultivar number No.221 present in Fig 7J and 7K were not validated for external datasets. (TIF) [file pone.0291105.s002.tif]

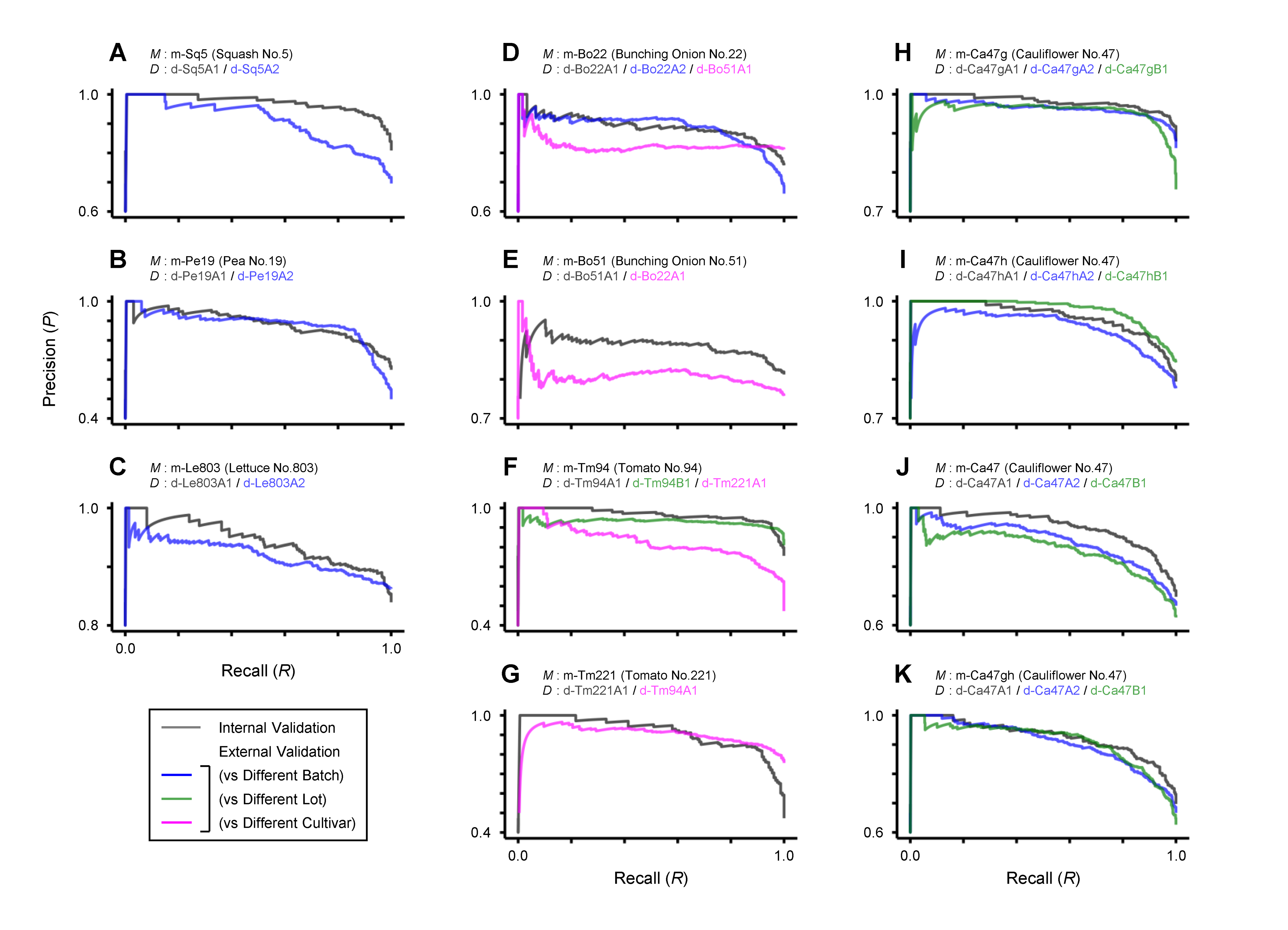

Supplement: S3 Fig — The figure is drawn in the same style as Fig 8. The vertical axis indicates precision (P) in place of relative precision in Fig 8. (TIF) [file pone.0291105.s003.tif]

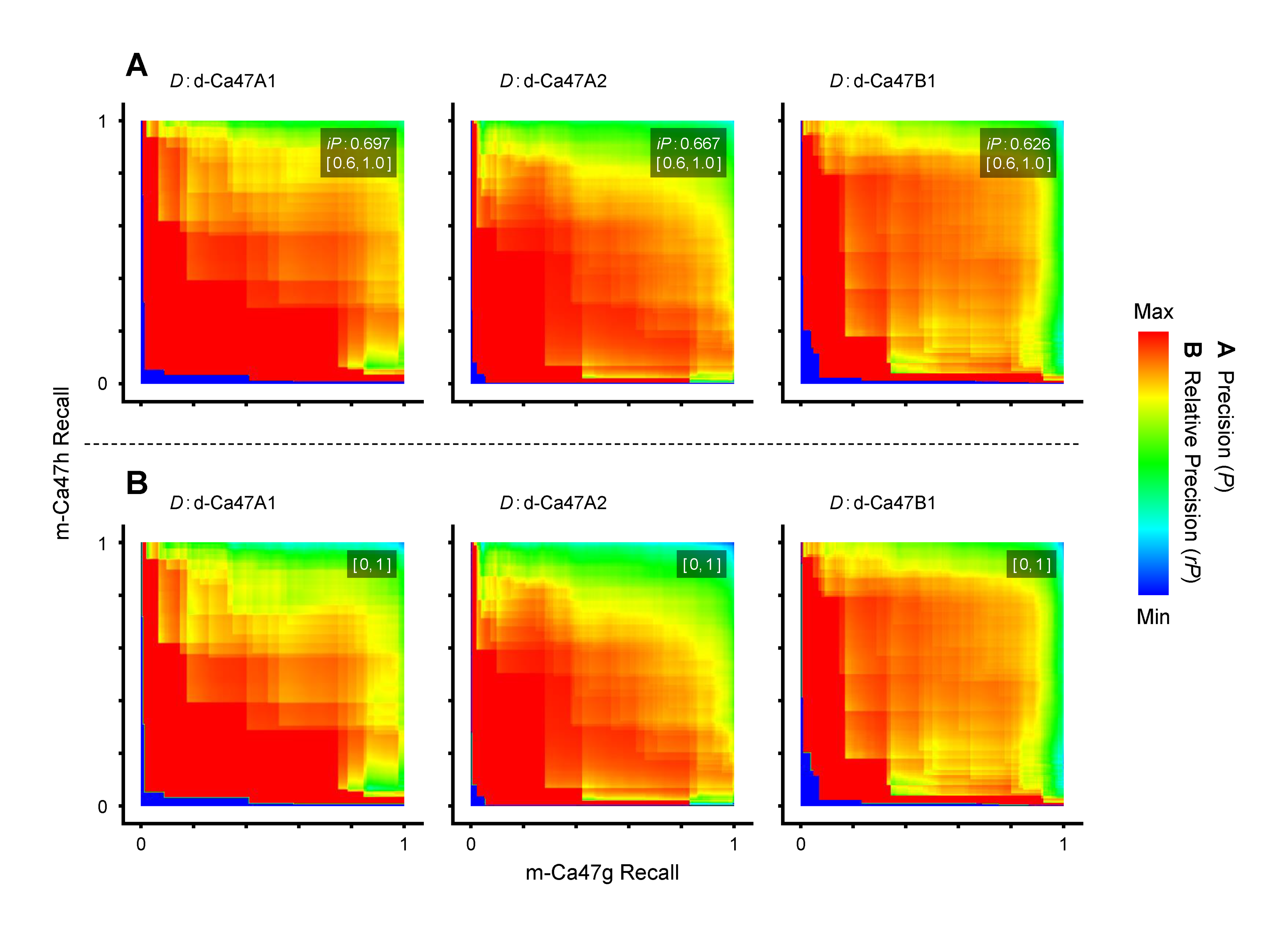

Supplement: S4 Fig — Curves (curved surfaces) representing precision-recall (PR) (A) and relative precision-recall (rPR) (B) relationships are drawn planarly in head maps. X- and Y-axes represent recall (R) in per-trait discrimination with m-Ca47g and m-Ca47h models, respectively. Color scales represent closed intervals of [0.6, 1.0] in (A) and [0, 1] in (B). Initial precision (iP) of the dataset D under analysis are indicated on upper right of each heat map in (A). Results for the dataset d-Ca47A1 are of internal, and the others of external validations. (TIF) [file pone.0291105.s004.tif]
