# Supplementary material for: Last-percent improvement in eligibility rates of crop seeds based on quality evaluation using near-infrared imaging spectrometry
Source: PLoS One. 2023 Sep 20;18(9):e0291105. doi: 10.1371/journal.pone.0291105 (PMC10511137; doi:10.1371/journal.pone.0291105)
Supplement: S1 File — (ZIP) [file pone.0291105.s005.zip › HOWTO.pdf]

Steps for retesting the procedures in S1 Fig.

Note:

- Running the script suggests discriminant models with acceptable performance, but the results will not necessarily be identical to those seen in the manuscript.
- Even if the conditions for deriving discriminant models are specified explicitly, partial regression coefficients, etc. will not be exactly identical to those seen in Fig 5. However, it does not make a big difference in terms of performance.

1. Unzip the S1 File and check that the files in the created folder are arranged as follows.

   ┬ data —— (.csv files) : including all datasets used in this study
   └ (.R files) : R script files written in plane text format

2. Place these files in your R working directory without changing their relative locations. Alternatively, change the working directory of running R to the folder containing these files.

3. (S1 Fig. Step 1: Dataset Preparation)
   Run the statement source("ST1_SpectralTransformation.R") from the console. This yields 12 spectra from each of 13 datasets with technical duplication (12 × 13 × 2 = 312 CSV files). It will take some time, so if necessary, comment out the unneeded datasets from lines 24–39 of the script to exclude from processing.

4. (S1 Fig. Steps 2–4: Deriving Candidate Discriminant Models)
   1) Open "ST2_ModelDerivation.R" script in a text editor. From lines 23–32, uncomment one dataset that is to be used for model derivation (d-Ca47A1 is selected by default). As script takes a long time to complete, make adjustments such as limiting the range of spectra to be processed ('specs', line 34) or reducing the number of iterations ('nIter', line 35).
   2) Run the statement source("ST2_ModelDeviation.R") from the console.
   3) A CSV file named "ST2_Results.csv" will be generated in the same folder as the script. Here, the "standard performance" and "AUC-PR/rPR" of each candidate model is shown row by row. To narrow down the models that can be adopted, open this file with Excel and try sorting the rows in descending order of the column val1.sP (or val2.sP), which represents the post-sorting precision as calculated by outer cross-validation.

Steps for retesting the procedures in S1 Fig.

5. (S1 Fig. Step 4: Re-derivation Test of Candidate Discriminant Models)
   1) Open "3_CheckSelected.R" script in a text editor. Edit lines 25–34 according to the original description. Dataset (string) and spectrum number (numeric) used for deriving discriminant models, and element numbers of selected wavebands (numeric vector) should be specified in a comma-separated-values format in this order.
   2) Run the statement source("ST3_CheckSelected.R") from the console.
   3) A CSV file named "ST3_Results.csv" will be generated in the same folder as the script. Check its content and decide the number of latent components to be used in PLS-DA.

6. (S1 Fig. after Step 4: Getting Details of Discriminant Models)
   1) Open "4_Predict.R" script in a text editor. Edit lines 25–93 according to the original description. Datasets for training and testing (string), spectrum number (numeric) used for deriving a discriminant model, the numbers of latent components (vector of length three), and element numbers of selected wavebands (vector of varying length) should be specified as an object of a list variable.
   2) Include all keys specifying crop cultivars to be processed in a vector variable 'keys' (l. 95).
   3) Run the statement source("4_Predict.R") from the console.
   4) Four CSV files with the prefix "ST4-" will be generated for each cultivar in the same folder as the script. Each file describe the following content:

      4-1: Performance metrics (standard and overall) of each model.
      4-2: SPRC and VIP for selected wavebands in each model.
      4-3: Discriminant scores for each seed in the internal validation
      4-4: Discriminant scores for each seed in the external validation
