# Supplementary material for: Last-percent improvement in eligibility rates of crop seeds based on quality evaluation using near-infrared imaging spectrometry
Source: PLoS One. 2023 Sep 20;18(9):e0291105. doi: 10.1371/journal.pone.0291105 (PMC10511137; doi:10.1371/journal.pone.0291105)
Supplement: S2 File — (ZIP) [file pone.0291105.s006.zip › HOWTO.pdf]

Steps to reproduce Fig 10.

Note:

- The program “HyperImageRegressor” is not matured enough and operation can become unstable. If it freezes, please forcibly terminate it from the task manager.
- The program does not support display scaling. When running with an HDPI (high dot per inch) monitor, please set the scaling to 100%.

1. Unzip the S2 File and check that the files in the created folder are arranged as follows.

```
──┬─ HyperImageRegressor (shortcut)
  ├─ bin ──┬─ model
  │        └─ (.exe, .dll, and .json files)
  └─ data ─── (.hyp files)
```

2. Double-click the “HyperImageRegressor” shortcut. If the program does not start, download and install the .NET6 runtime from the URL below.
   https://dotnet.microsoft.com/ja-jp/download/dotnet/thank-you/runtime-aspnetcore-6.0.20-windows-x64-installer

3. Open the “data” folder. Then drag and drop one of the .hyp files onto the “HyperImageRegressor” Main Window.

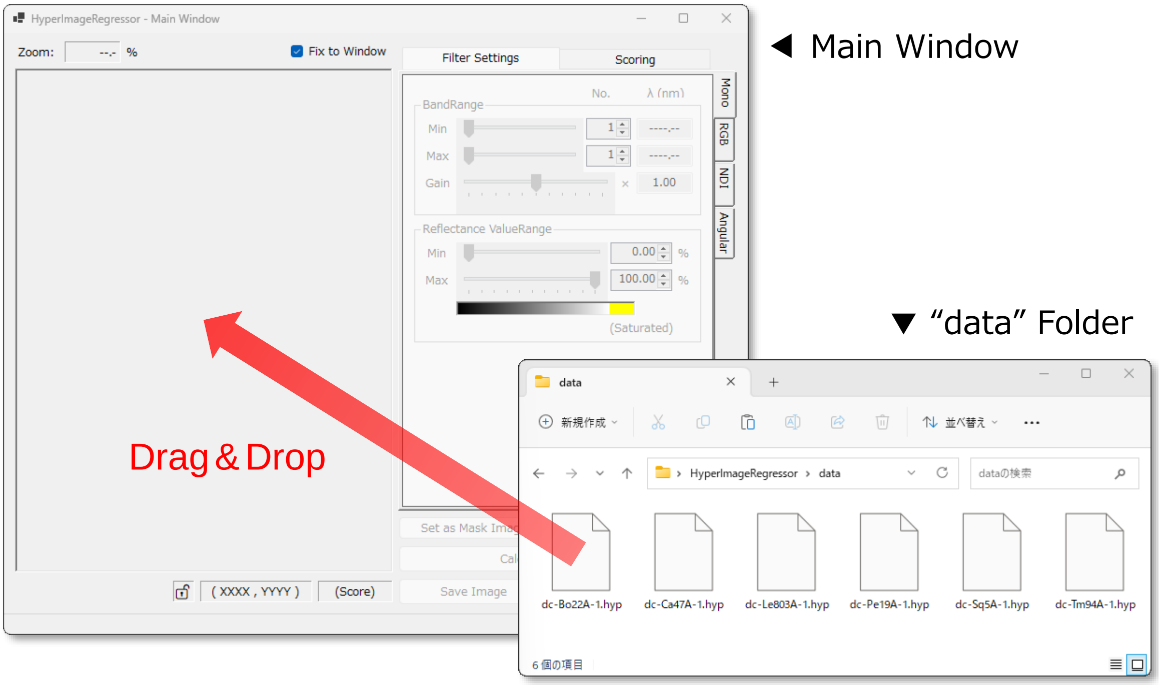

4. Filter Settings for Isolating Seed Areas
   1) Adjust the track bars① so that the background becomes completely black. Click the “Calculate & Redraw” button② to reflect the settings and refresh the image. A high “Gain” setting③ is recommended to ensure that no noise remains in the background.
   2) Wavebands suitable for separating seed areas from the background can be explored by clicking the “Show Plot Window” button④. The reflectance spectrum at the cursor coordinates on the image is displayed in the Plot Window.
   3) Click the “Set as Mask Image” button⑤ to complete this step.

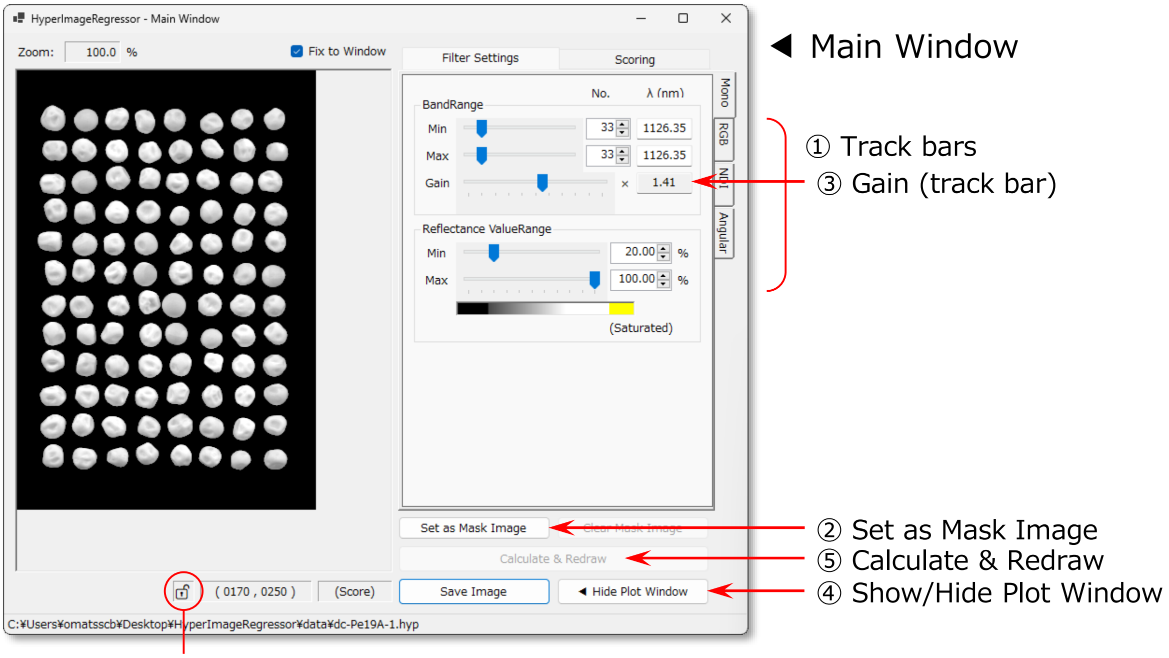

Cursor Lock Status Icon
The coordinate can be locked/unlocked by clicking on the displayed image.

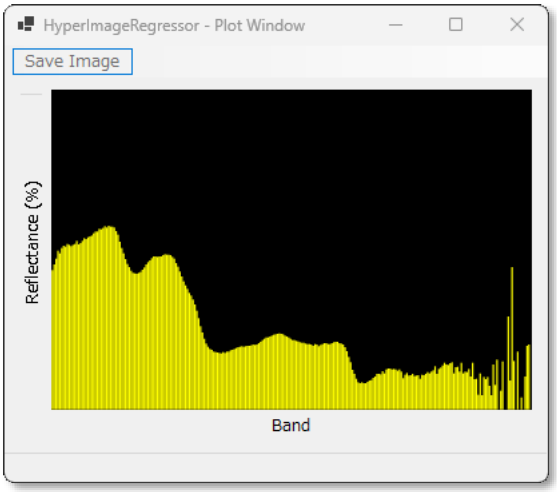

▲ Plot Window

4. Generating Score Image

   1) Switch the horizontal tab menu to “Scoring”①.

   2) Find the "Preset" drop-down menu② and select the discriminant model that matches the loaded crop cultivar.

   3) Click the “Calculate & Redraw” button③ to complete this step.

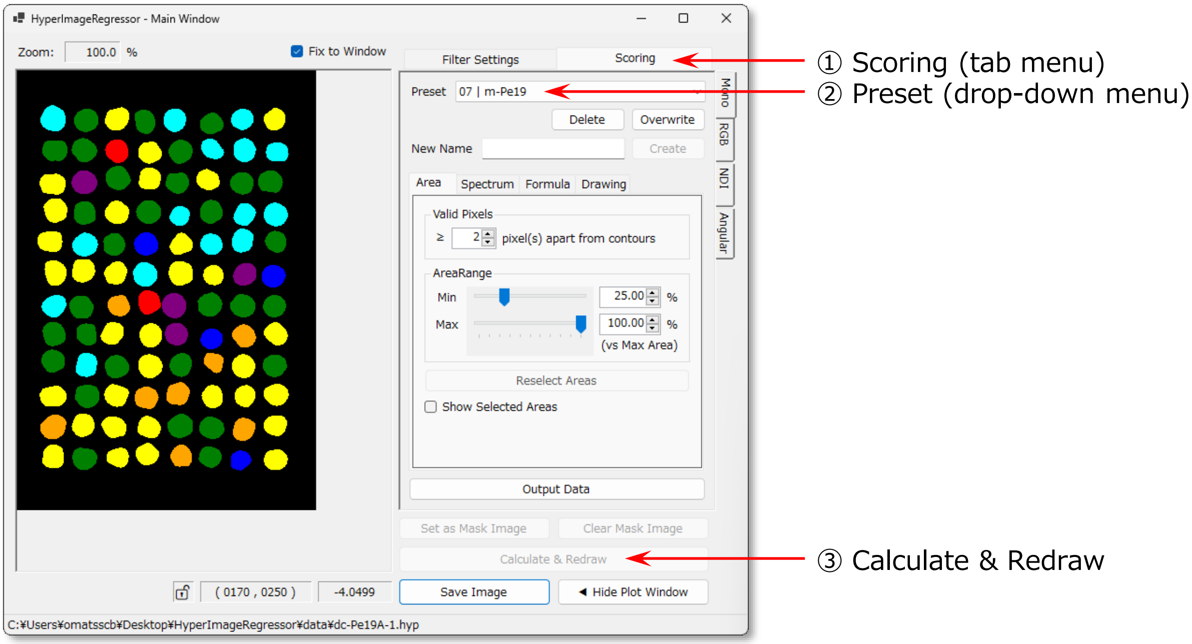

▲ Main Window
